# Uncovering how selected potent bacteriocins reshape the broiler chicken gut microbiome in a PolyFermS continuous in vitro model

**DOI:** 10.1186/s40104-026-01431-w

**Published:** 2026-06-12

**Authors:** Amal Mamjoud, Séverine Zirah, Sylvie Rebuffat, Éric Biron, Ismail Fliss

**Affiliations:** 1 Unit Molecules of Communication and Adaptation of Microorganisms (MCAM), UMR 7245 CNRS- MNHN, Paris, 75005 France; 2 Food Science Department, Food and Agriculture Faculty, Université Laval, Quebec, QC G1V 0A6 Canada; 3 Institute of Nutrition and Functional Foods, Université Laval, Quebec, QC G1V 0A6 Canada; 4 Faculty of Pharmacy, Université Laval and Centre de Recherche du CHU de Québec-Université Laval, Quebec, QC G1V 0A6 Canada

**Keywords:** Antibiotics, Bacteriocins, Microbiota, Microcin J25, Nisin, Pediocin, PolyFermS, Poultry

## Abstract

**Background:**

Bacteriocins are promising alternatives to antibiotics in poultry production, offering pathogen control with minimal disruption to gut microbiota and reduced risk of resistance dissemination. This study compared the impact of three bacteriocins—microcin J25, nisin Z, and pediocin PA-1—against bacitracin as a positive control, specifically evaluating their effects on gut microbiota composition and metabolic activity. The study utilized the PolyFermS continuous fermentation model to simulate chicken caecal conditions.

**Results:**

16S rRNA sequencing revealed that bacitracin and nisin Z significantly altered the microbiota composition, reducing key families such as Lactobacillaceae and Ruminococcaceae, while microcin J25 and pediocin PA-1 had negligible effects. Short-chain fatty acid analysis revealed a significant time-dependent decrease in butyrate levels following nisin Z and bacitracin treatments, with the most pronounced reduction observed at 48 h post-injection. Conversely, microcin J25 and pediocin PA-1 maintained stable profiles. Untargeted LC-MS metabolomics indicated a marked metabolic shift under nisin Z treatment, including increased amino acids and cyclic dipeptide levels, while microcin J25 had minimal impact. Stability assays confirmed that microcin J25 remained active up to 24 h, whereas nisin Z and pediocin PA-1 lost antimicrobial activity rapidly.

**Conclusions:**

These findings support the selective use of bacteriocins as alternatives to antibiotics in poultry farming, with microcin J25 demonstrating the most favorable profile for microbiota preservation and metabolic stability. Future research will focus on in vivo trials in broiler chickens to confirm these findings and evaluate the practical application of microcin J25 in a production environment.

**Supplementary Information:**

The online version contains supplementary material available at 10.1186/s40104-026-01431-w.

## Introduction

The poultry industry plays a crucial role in global food production and holds significant economic importance, providing an cost-effective source of proteins in human diets [1]. Understanding the poultry caecal microbiome and its modulation is crucial for both optimizing poultry production and controlling pathogenic bacteria [2]. The ceca provide an ideal environment for bacterial fermentation, being the most densely populated section of the gastrointestinal tract, characterized by a slow digestive transit time of 12–24 h [3, 4]. It also may be colonized by harmful pathogens like *Salmonella* and *Campylobacter* [5]. Consequently, the caecum's dual role in both digestion and pathogen control has made it a focal point for research on chicken microbiota, aiming to enhance poultry health, improve feed efficiency, and develop effective strategies for disease prevention [6].

Antibiotics have been extensively used in poultry production, to protect from infections and promote growth. However, the abuse of many antibiotics generated a burden of multi-resistant bacteria [7, 8]. Solutions with less impact on both antibiotic resistance dissemination and gut microbiota diversity, such as nutrients, probiotics, prebiotics, natural antimicrobial peptides and other feed additives are garnering interest to improve chicken health, immune function, growth performance, and product quality (meat and egg) [9, 10]. Among antimicrobial peptides, bacteriocins have shown great potential in controlling pathogenic bacteria in livestock; their targeted mechanisms and rapid action may significantly reduce the likelihood of developing the widespread resistance typically associated with conventional antibiotics [11, 12]. These peptides are ribosomally synthesized by a diverse range of bacterial species, exhibiting a broad spectrum of activity—ranging from narrow to wide—as well as highly varied structural profiles and mechanisms of action. Bacteriocins are generally categorized into three primary classes based on their size and stability: class I consists of post-translationally modified peptides; class II includes heat-stable, unmodified peptides; and class III comprises larger, heat-labile proteins, often possessing bacteriolytic properties [13].

The impact of bacteriocins on the modulation of colonic microbiota has been studied in humans and animals. The first approved bacteriocin in the food and veterinary industry is nisin, a lanthipeptide which exerts a dual mode of action, through binding to lipid II, thereby preventing cell wall synthesis and forming transmembrane pores [14].

The efficacy of these bacteriocins—nisin, pediocin PA-1, and microcin J25—extends beyond simple pathogen reduction to the modulation of the caecal microbiota, a critical reservoir for both commensal fermentation and enteric pathogens. In poultry and swine, nisin and microcin J25 specifically target the caecal environment to suppress the proliferation of *Salmonella* and *E. coli* while simultaneously improving feed utilization [15–17]. For instance, pediocin PA-1 disrupts the mannose phosphotransferase system (Man-PTS) to induce pore formation in sensitive strains [18, 19], yet research suggests that its administration can increase weight gain in animal models without causing significant dysbiosis in the broader intestinal or caecal populations [20]. Similarly, the lasso peptide microcin J25 maintains its anti-*Salmonella* activity even under the complex conditions of the porcine colon and caecum, effectively reducing localized inflammation while preserving the microbial balance necessary for growth [16, 21, 22]. In a recent study, we used a batch fermentation model to screen the effects of various classes of bacteriocins and antibiotics at different concentrations on the chicken caecal microbiota composition and metabolic activity [23]. Among the bacteriocins tested, nisin Z induced the most extensive alterations to the caecal microbiota composition, followed by a synthetic analogue of pediocin PA-1 (M31L) designed to be resistant to oxidation with biological activity equal to or greater than the native peptide [18]. In contrast, microcin J25 did not significantly modify the microbiota composition.

In this study, a more in depth comprehensive and temporal assessment was performed to evaluate the impact of three bacteriocins with different structures and mechanisms of action, namely nisin Z, pediocin PA-1, and microcin J25 on modulation of the microbiota, using the PolyFermS fermentation system to replicate poultry caecal microbiota, in comparison with bacitracin. The PolyFermS continuous fermentation model addresses the limitations of batch fermentations, such as nutrient depletion, absence of pH control, thus permitting to produce a stable microbiota without time limitation [24]. The microbiota composition was followed by 16S sequencing while the microbial metabolic activity was assessed using short-chain fatty acid quantification and LC-MS based untargeted metabolomics. In addition, the stability of bacteriocins over time was assessed through antimicrobial activity measurements and LC-MS/MS profiling.

## Materials and methods

### Antimicrobial production

Microcin J25 was produced by fermentation using recombinant *E. coli* MC4100 harboring the plasmid pTUC202 [25] and purified from culture supernatants by solid-phase extraction (SPE) followed by reverse-phase high-performance liquid chromatography (RP-HPLC) [26]. Pediocin PA-1 was produced using a standard solid-phase peptide synthesis (SPSS) protocol and purified using RP-HPLC [18]. Nisin Z was purified from a commercial nisin solution from Nissen^®^-S10, Fromagex, Canada, using the lasting out procedure as described by Bennett et al. [27]. The structural identity and biological equivalence of these peptides were confirmed by LC-MS, ensuring that the mode of production (recombinant, synthetic, or commercial) did not affect their standardized antimicrobial activity (Fig. S1). The antibiotic bacitracin was obtained from Sigma-Aldrich.

The concentrations of the antibacterial compounds used in the present study was 250 times the minimum inhibitory concentration against their respective standard indicator strains, based on our previous study [23]. The minimal inhibitory concentration (MIC) values as well as the susceptible strains used to confirm the inhibition activity of the different bacteriocin preparations are listed in Table S1.

### Fermentation medium

The modified Viande Levure (VL) medium (composition in Table S2) was used to reproduce the conditions of the poultry caeca. This medium was supplemented with Tween 80, bile salts (0.4 g/L), mucin (2.0 g/L), fructooligosaccharides (2.5 g/L), pectin (2.5 g/L), along with essential minerals and vitamins based on the microbiota growth medium described in [28]. The pH of the medium was adjusted to 6.0 using 5 mol/L NaOH.

### Caecal collection, immobilization, and bead colonization

In the anaerobic chamber, the caecal contents from the four birds were pooled and squeezed into a sterile Stomacher bag. The material was mixed with 0.1% peptone water reduced with 0.05% cysteine to create a 10% (w/v) slurry and homogenized for 2 min at 200 × r/min (Seward model 400, Norfolk, UK). The liquid part was recovered in a 50-mL Falcon tube and centrifuged for 1 min at 700 r/min. The entire immobilization procedure was conducted under anaerobic conditions. Supernatant from the caecal slurry was used to inoculate a solution of 2.5% (w/v) gellan and 0.25% (w/v) xanthan at 2%. Gel beads were formed by adding the gum solution to sterile sunflower oil maintained at 40 °C under constant magnetic stirring. The stirring speed was calibrated to achieve target bead diameters within the 1–2 mm range. After removing the oil, a 0.1 mol/L CaCl_2_ solution was added to harden the beads and let under low stirring for 30 min. Then the beads were washed with a 0.27 mol/L KCl + 0.03 mol/L CaCl_2_ solution. Beads with a diameter of 1–2 mm were selected by wet sieving [29].

The immobilized chicken caecal microbiota beads (150 g) were transferred to a glass bioreactor (Multifors, Infors AG, Bottmingen, Switzerland) holding 350 mL of sterile fresh nutritive medium. To ensure initial bead colonization, the whole culture medium was withdrawn manually 20 h and 26 h after inoculation of the fermenter with the beads and replaced with a sterile fresh medium. After the first colonization of caecal beads, the reactor was run for the first 20 days to stabilize fecal bead colonization. Anaerobiosis was generated by continuous flushing of the headspace of the reactor and fermentation medium with pure filter sterile CO_2_. The temperature was controlled at the chicken body temperature of 41 °C, and the pH was kept at 6.0 by automatic addition of 2.5 mol/L NaOH [28].

### Experimental design of continuous fermentation for caecal microbiota

**Fig. 1:**
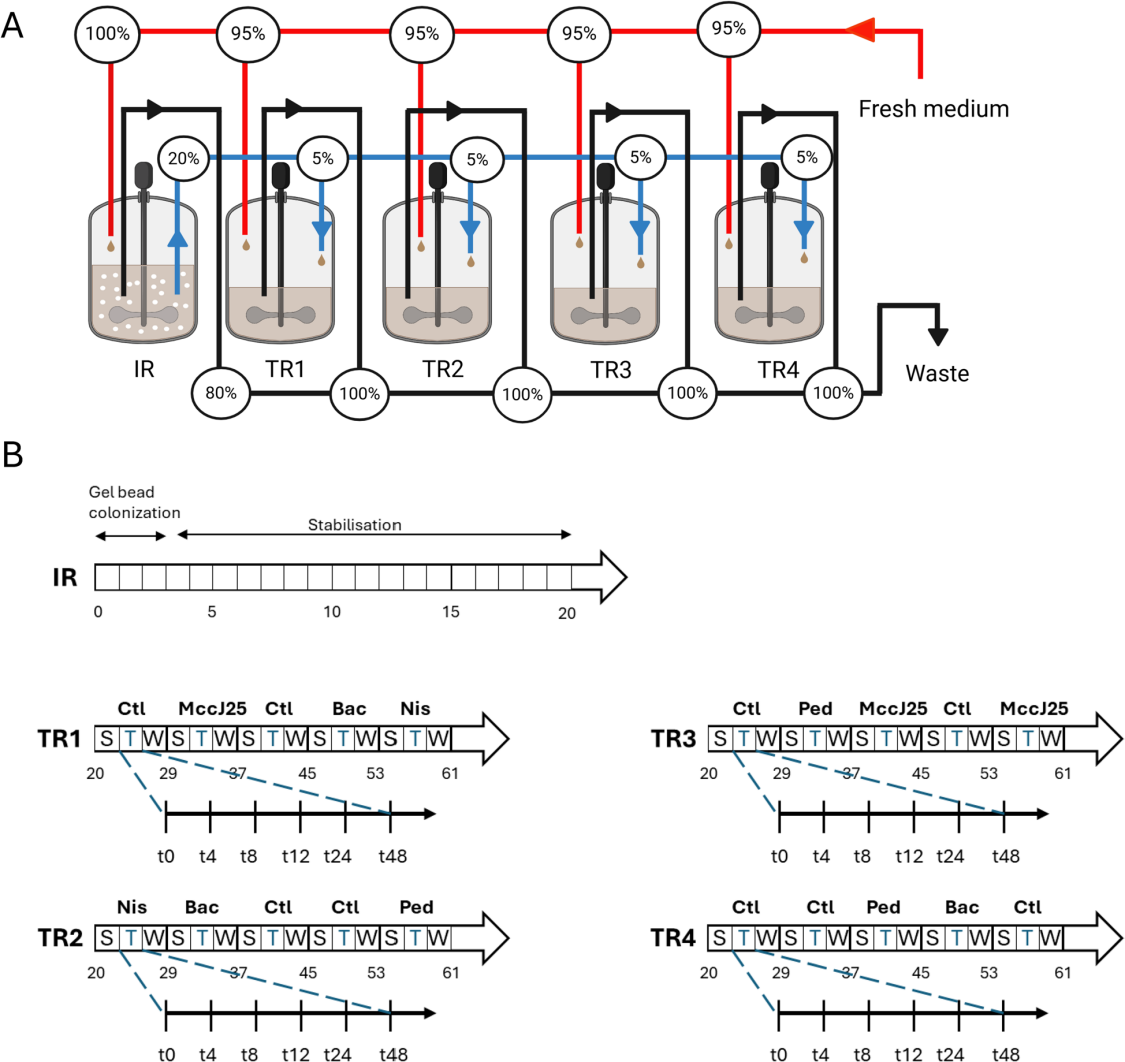
Experimental design of continuous fermentation. **A** Experimental conditions tested in the PolyFermS model mimicking the chicken caecal microbiota. Inoculum reactor (IR) containing caecal microbiota immobilized in gel beads. Test reactors (TR) continuously inoculated with 5% fermentation effluent from the IR and 95% nutritive media. **B** Experimental plan for the treatment’s injection on the TR. After each cycle, the TR are washed and sterilized. Stabilization period (S); Test period (T); Washing (W)

The continuous fermentation in vitro model of the poultry caeca in this study utilizes the PolyFermS platform, as described by Asare et al. [28]. This two-stage system consists of one 500 mL inoculum reactor (IR) and four 250 mL test reactors (TR) (Multifors, Infors AG, Bottmingen, Switzerland). The reactors were kept at 41 °C and pH 6.0 under anaerobic conditions, achieved through continuous flow of pure CO_2_ through the medium. After stabilization of the IR, the TR were connected to the system. The IR received a continuous feed of fresh nutrient medium, which was then used to supply the test reactors TR1, TR2, TR3, and TR4. The feed composition for each TR consisted of 5% inoculum broth from the IR and 95% fresh medium. The continuous flow rate was set to 8.4 mL/h, corresponding to a mean retention time of 24 h, chosen to simulate the infrequent emptying of the caeca in poultry. This PolyFermS design enables the generation of similar microbiota composition and activity across all TRs, as they are inoculated with the same microbiota produced in the IR. The experimental design utilized the four TRs in three consecutive cycles to obtain *n* = 3 independent replicates per treatment. Between cycles, TRs were emptied and sterilized to prevent carry-over effects, while the IR remained in continuous steady-state to provide a consistent inoculum. This approach allowed for a randomized block design where each treatment was tested across different time blocks, ensuring that the observed effects were not due to temporal fluctuations within the system (Fig. 1).

### Propidium-monoazide-coupled quantitative polymerase chain reaction

The stability of the reactors was assessed by quantifying the major bacterial species constituting the chicken colonic microbiota using propidium-monoazide-coupled quantitative polymerase chain reaction (PMA-qPCR). Samples collected from the reactors were treated with propidium monoazide (PMA dye, Biotium, Inc., Hayward, CA, USA). Briefly, 1.25 μL of 20 mmol/L PMA solution and 800 μL 1 mmol/L peptone water were added to 200 μL of sample collected. Samples were kept for 5 min at room temperature in the dark with occasional Vortex mixing, then exposed to LED light (Biotium Inc., Fremont, CA, USA) for 15 min [30]. DNA extraction was carried out following the protocol detailed in the following section.

The quantitative PCR (qPCR) was performed in MicroAmp R^©^ Fast Optical 96-Well Reaction Plates with Barcode (Life Technologies Inc., Burlington, ON, Canada) on an Applied Biosystem 7500 instruments (Applied Biosystems, Streetsville, ON, Canada). The primers used in this study are given in Additional file 1 (Table S3). Each reaction was run in triplicate in a 96-well reaction plate sealed and maintained at a final volume of 15 μL. The qPCR reaction and thermal cycling amplification procedure followed was previously described in [31]. Absolute quantification of gene copy numbers utilized standard curves obtained through droplet digital PCR (ddPCR) on the IBIS (Institute for Integrative Systems Biology, Laval University) sequencing platform, as described previously [32].

### DNA extraction and 16S rRNA gene sequencing

The microbial composition of the reactors was assessed by quantifying the major bacterial species constituting the poultry colonic microbiota. Samples collected from the reactors were treated with propidium monoazide (PMA dye, Biotium, Inc., Hayward, CA, United States). Enzymatic lysis of DNA was performed using proteinase K (20 µg), lysozyme (20 mg), and mutanolysin (50 U), followed by extraction with the QIAamp PowerFecal Pro DNA Kit (Qiagen, Hilden, Germany). Concentration was assessed using a NanoDrop 1000 spectrophotometer (Thermo Scientific, Wilmington, DE, USA). The 16S rRNA V3–V4 region was amplified using primer pairs 341F–805R and sequenced at the Centre de recherche du CHU de Québec-Université Laval using Illumina MiSeq paired-end technology.

Sequences were analyzed in the Ubuntu terminal using the QIIME2-amplicon 2024.10 pipeline [33]. Samples were demultiplexed using q2-demux, truncation and denoising was performed using DADA2 [34]. Chimeric sequences were identified and removed using UCHIME [35]. Taxonomy was assigned by using the q2-feature-classifier classify-sklearn against pre-trained classifier Silva reference database [36]. Data were visualized and analyzed using R (4.4.1). A phyloseq data object was created using the phyloseq package (1.50.0). The Shannon diversity index was used to compare treatment effects. Beta diversity was evaluated by principal coordinate analysis (PCoA) based on Bray–Curtis distance [16].

### Quantification of volatile fatty acids (VFAs) by gas chromatography coupled with flame ionization detector (GC-FID)

Fermentation samples were subjected to VFA analysis using GC-FID, following the method described by Roussel et al. [37]. The extraction process used methyl tert-butyl ether as the solvent. The analysis quantified a range of VFAs, including acetate, propionate, butyrate, isobutyrate, valerate, isovalerate, and hexanoate.

### Liquid chromatography coupled with mass spectrometry (LC-MS)

Fermentation samples were suspended in an equal volume of acetonitrile to achieve a concentration of 50% acetonitrile/water (v/v). The homogenate was then centrifuged at 14,000 × *g* and 4 °C for 8 min, before being analyzed by UPLC (Ultimate 3000 RSLC, Thermo Scientific, Waltham, MA, USA) coupled with a high-resolution electrospray ionization-quadrupole-time of flight (ESI-Q-TOF) mass spectrometer (Compact, Bruker Daltonics, Billerica, MA, USA). Metabolite separation was achieved on an Acclaim RSLC Polar Advantage II column (PA2) (2.2 μm, 2.1 mm × 100 mm, 120 Å, Thermo Fisher Scientific, Waltham, MA, USA) at a flow rate of 300 μL/min using gradients of solvent A (ultra-pure water/0.1% formic acid) and solvent B (HPLC-MS grade acetonitrile/0.08% formic acid). LC-MS experiments were performed in positive ion mode in the range *m/z* 50–1,000. In addition, data-dependent LC-MS/MS using collision-induced dissociation (CID) analyses on the most intense ions and targeted LC-MS/MS using multiple reaction monitoring mode on features of interest were acquired. A quality control (mix of every sample) and a blank with extraction solvent sample were injected every ten runs to check repeatability of the separation and absence of cross-contamination, respectively.

Relative quantification of microcin J25, nisin Z, and bacitracin was achieved by extracting the peak areas of the extracted ion chromatograms of interest. The MS spectra were acquired in positive ion mode in the mass range *m/z* 50–1,800, using the following gradient of solvent A (milliQ water + 0.1% formic acid) and solvent B (HPLC-MS grade acetonitrile + 0.08% formic acid) over a total run time of 21 min. Data-dependent LC-MS/MS data were acquired in positive ion mode in the mass range *m/z* 50–1,800, using CID with collision energy calculated from *m/z* and charge states. The data were processed with Data Analysis 4.4 (Bruker Daltonics).

The raw data were converted to mzXML format and analyzed using R version 4.4.1. The package XCMS (4.4.0) [38], which enables peak detection, retention time alignment, and peak matching was used to produce a multivariate matrix containing feature intensities in every samples. The CentWave peak detection method was configured with a maximally tolerated *m/z* deviation of 10 ppm. The generated multivariate matrix was subjected to multivariate data analysis using the package mixOmics (6.30.0) [39]. Principal component analysis (PCA) and partial least squares discriminant analysis (PLS-DA) were performed. The latter was combined with a variable importance in projection (VIP) analysis to identify compounds with the highest discrimination in the prediction models, which were classified according to their VIP score (VIP score threshold = 2). The raw formulas for the most discriminant features were calculated using DataAnalysis 4.4 (Bruker Daltonics).

The XCMS peak list was exported as an.mgf file and uploaded to SIRIUS 6.0.7 [40] and SIRIUS CSI: FingerID [41], and CANOPUS [42–44] analyses were performed and validated by manual inspection of the MS/MS data. In parallel, the dataset was processed using the GNPS2 feature-based molecular networking workflow (accessed on 2025-05-25) to support spectral annotation and molecular relationship visualization.

### Correlation analyses between microbiota composition, VFAs and metabolomics data

Correlations were calculated between two different data sets, i.e., bacterial genera abundances (microbiota) and metabolic profiles, including both VFAs and selected metabolites form the untargeted metabolic analysis using the cor.test function from stats 4.4.1. Thereafter, Spearman method was applied for multiple testing correction. Adjustment for multiple comparisons was carried out with Benjamini & Hochberg method. Correlation results were plotted as network with the Igraph 2.1.4.

### Functional prediction

Metabolic pathways were predicted from 16S rRNA gene sequences using the PICRUSt2 pipeline (v2.5.2) [45]. Pathway abundances were normalized and annotated using the MetaCyc database [46]. Subsequent statistical analysis and visualization were performed in R (v4.3.0) using the ggpicrust2 package (V2.5.10) [47]. To facilitate a mechanistic interpretation, significant pathways were manually categorized into functional themes, including energy metabolism, amino acid biosynthesis, and structural integrity.

### Antimicrobial activity assays and microtitration assays

The inhibitory activity of the bacteriocins and the control antibiotic bacitracin in the fermented broth was determined qualitatively by the agar well diffusion assay, as described by Bédard et al. [18]. Briefly, 80 μL of samples were added into wells punched out in appropriate media (25 mL) seeded with 250 μL of overnight culture of indicator strains. Inhibition zones were measured after an 18-h incubation at 30 °C for* Listeria ivanovii* and 37 °C for *Salmonella enterica*.

Quantitative determination of inhibitory activity in the fermented broth was carried out as described by [48]. Briefly, two-fold serial dilutions of fermented broth including bacteriocins and bacitracin (125 μL) were prepared in an appropriate medium in a clear 96-well flat-bottom microtiter plate. Indicator strains were diluted to 10^5^ CFU/mL, from which 50 μL were added to each well. The microtiter plates were incubated for 24 h under appropriate conditions, and optical densities were recorded at 595 nm (Infinite M200, Tecan, Switzerland). The concentration of bacteriocins was calculated using the known minimum inhibitory concentration (MIC) and the number of dilutions performed.

### Statistical analysis

Statistical analyses were performed in version R 4.4.1. All statistical comparisons were performed with the package rstatix (0.7.2). Shapiro–Wilk test was performed to assess normality. Kruskal–Wallis test followed by Dunn’s post hoc test or the Wilcoxon rank-sum test were used for non-normal distributions. Adjustment for multiple comparisons was carried out with Benjamini & Hochberg method. To evaluate the effects of collection time and treatment group on microbiota community composition, Bray–Curtis distance matrices were compared using PERMANOVA. Finally, all graphics were generated with the packages ggplot2 (3.5.2) and ggpubr (0.6.1). Differential abundance analysis was performed within the QIIME 2 pipeline (version 2024.10) using the qiime composition ancombc plugin on non-rarefied feature tables collapsed at taxonomic level 6 (genus level). Analyses were performed separately for each time point (t0, t12, t24, t48), comparing treatments against the Control. The Benjamini–Hochberg FDR was applied (*q* < 0.05). Taxon labels were cleaned to retain the most specific identified level (e.g., “Unclassified Lachnospiraceae”) for visualization. To explore the relationships between microbial shifts and functional outputs, Spearman’s rank correlations were calculated between bacterial genus abundances and metabolic profiles (VFAs and untargeted metabolites) using the cor.test function in R 4.4.1. Only correlations with a significant *P*-value after Benjamini-Hochberg (FDR) adjustment (*q* < 0.05) were retained for further analysis. These significant interactions were visualized as a multi-omics network using the igraph 2.1.4 package, where nodes represent taxa or metabolites and edges represent the strength of the association.

## Results

### Bacteriocin production, purification and activity

The identity and purity of all bacteriocins were evaluated using liquid chromatography–high-resolution mass spectrometry (LC-HRMS), which demonstrated purity levels exceeding 95% (Fig. S1).

### Impact of treatments on the poultry intestinal microbiota composition

After 20 d, the microbiota in the IR reached a stable composition similar to that determined in the original inoculum (Fig. S2). The changes in microbial composition in response to the added antimicrobials were assessed through 16S rRNA gene sequencing analysis at 0, 12, 24 and 48 h. To monitor the stability of the system, VFAs were quantified periodically throughout the entire fermentation period (Fig. S3).

**Fig. 2:**
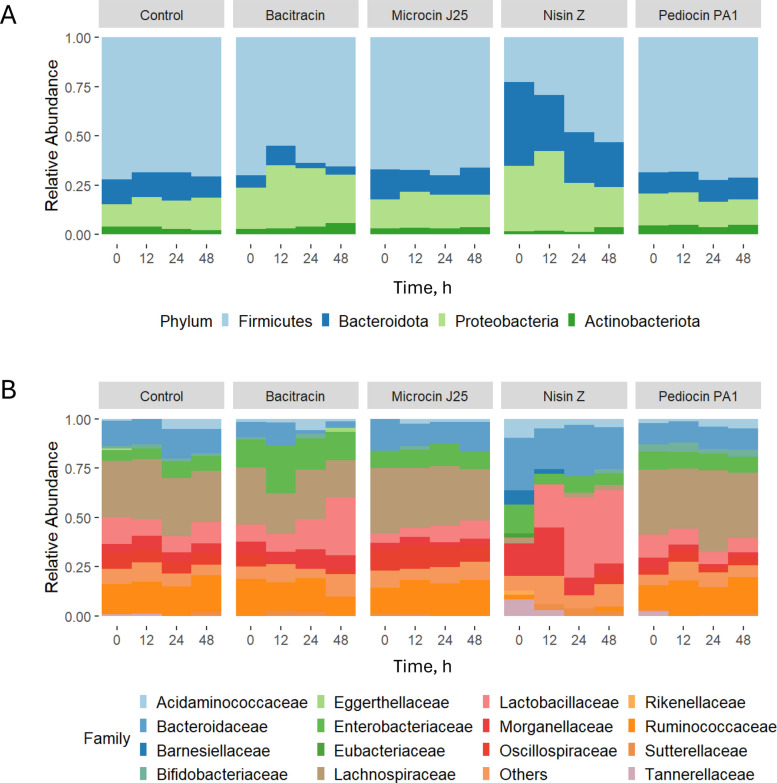
Chicken caecal microbiota composition during in vitro assay. Mean relative abundances of the predominant bacterial phyla (**A**) and families (**B**). Taxa with a mean relative abundance < 1% were filtered out, all remaining genera outside the top 20 were grouped as 'Others'. The control reactors received no antimicrobial agents and served as the baseline for comparison

In the control, the predominant bacterial phyla detected over 48 h of continuous fermentation were Firmicutes (72%), Bacteroidota (10%) Proteobacteria (12%) and Actinobacteriota (6%) (Fig. 2A). In the presence of bacitracin, Proteobacteria increased from 0 to 48 h with a peak at 12 h, Firmicutes decreased after 12 h and Bacteroidota after 24 h. Nisin Z had an immediate effect on the relative composition of the caecal microbiota. At 0 h after processing, Firmicutes were drastically reduced, while Bacteroidota and Proteobacteria increased. By 48 h, the phyla abundance tended to be similar to that of the control. In the presence of microcin J25 and pediocin PA-1, the phyla compositions were comparable to the control during the 48 h of ceacal fermentation.

**Fig. 3:**
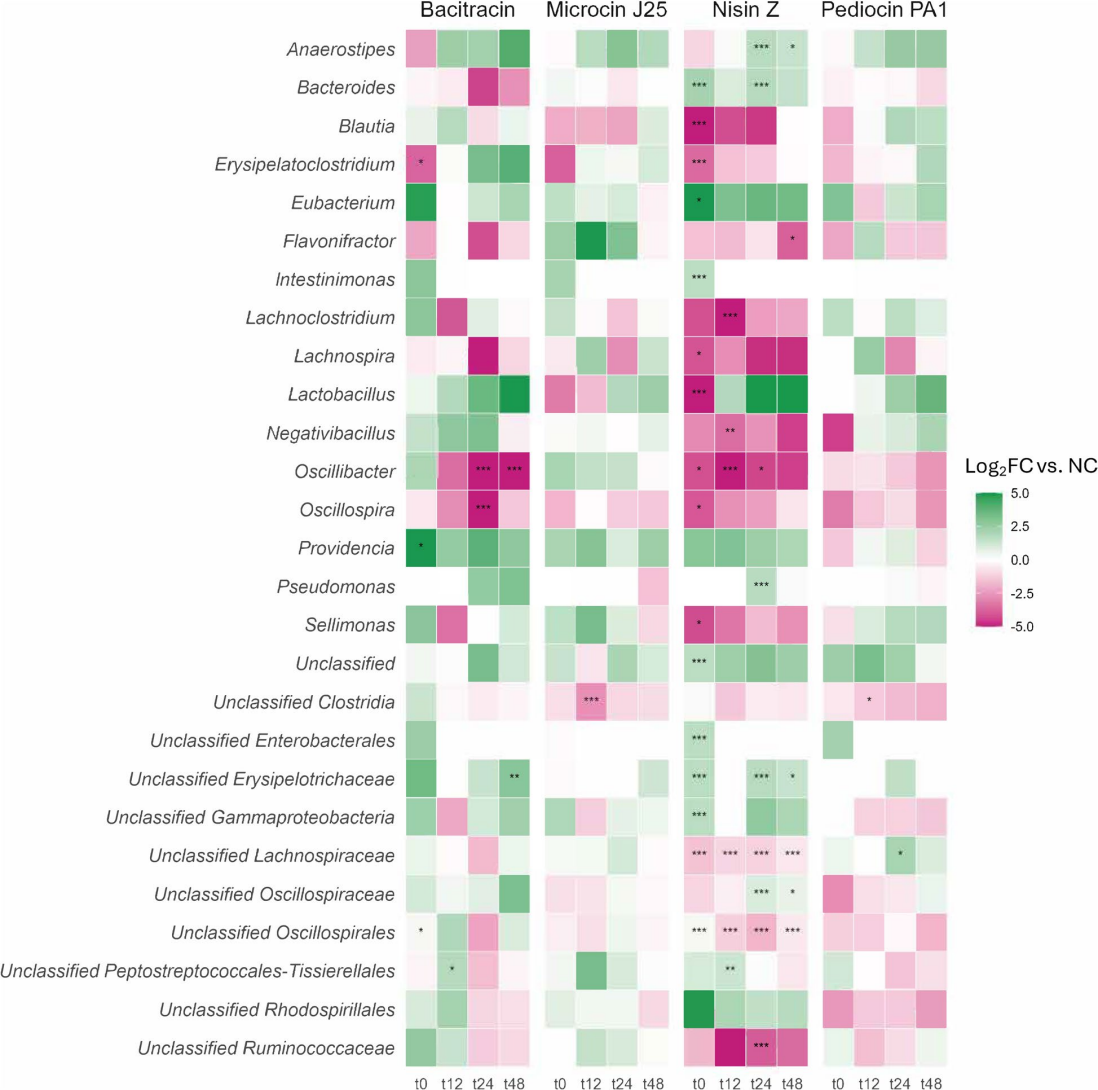
Differential abundance of bacterial genera across antimicrobial treatments. Bars represent log_2_ fold changes in relative abundance compared to the negative control (NC), as determined by ANCOM-BC analysis (FDR, adjusted *P* < 0.05). Taxa labeled as “Unclassified” correspond to genera with ambiguous assignments within higher taxonomic ranks

In terms of bacterial family composition, the control sample showed stable microbial communities, with minimal changes in relative abundances over time. Adding bacitracin resulted in a decrease in Bacteroidaceae together with an increase in Enterobacteriaceae. By 48 h, an increase in Lactobacillaceae was observed. Microcin J25 and pediocin PA-1 induced only a minor effect on the composition of the poultry caecal microbiota. Microcin J25 induced a transient decrease in Lactobacillaceae at 0 and 12 h post-treatment, whereas Enterobacteriaceae populations remained unaffected. Pediocin PA-1 caused a decrease in Morganellaceae after 24 h. The most pronounced effect was observed in the presence of nisin Z. At t0, a reduction in the relative abundance of several families was observed, in particular Lactobacillaceae, Ruminococcaceae, and Lachnospiraceae. Lactobacillaceae recovered 12 h after the addition of nisin Z (Fig. 2B). Differential analysis of bacterial abundances at the genus level (Fig. 3) revealed distinct signatures in response to nisin Z, with a significantly lower relative abundance of several taxa.

**Fig. 4:**
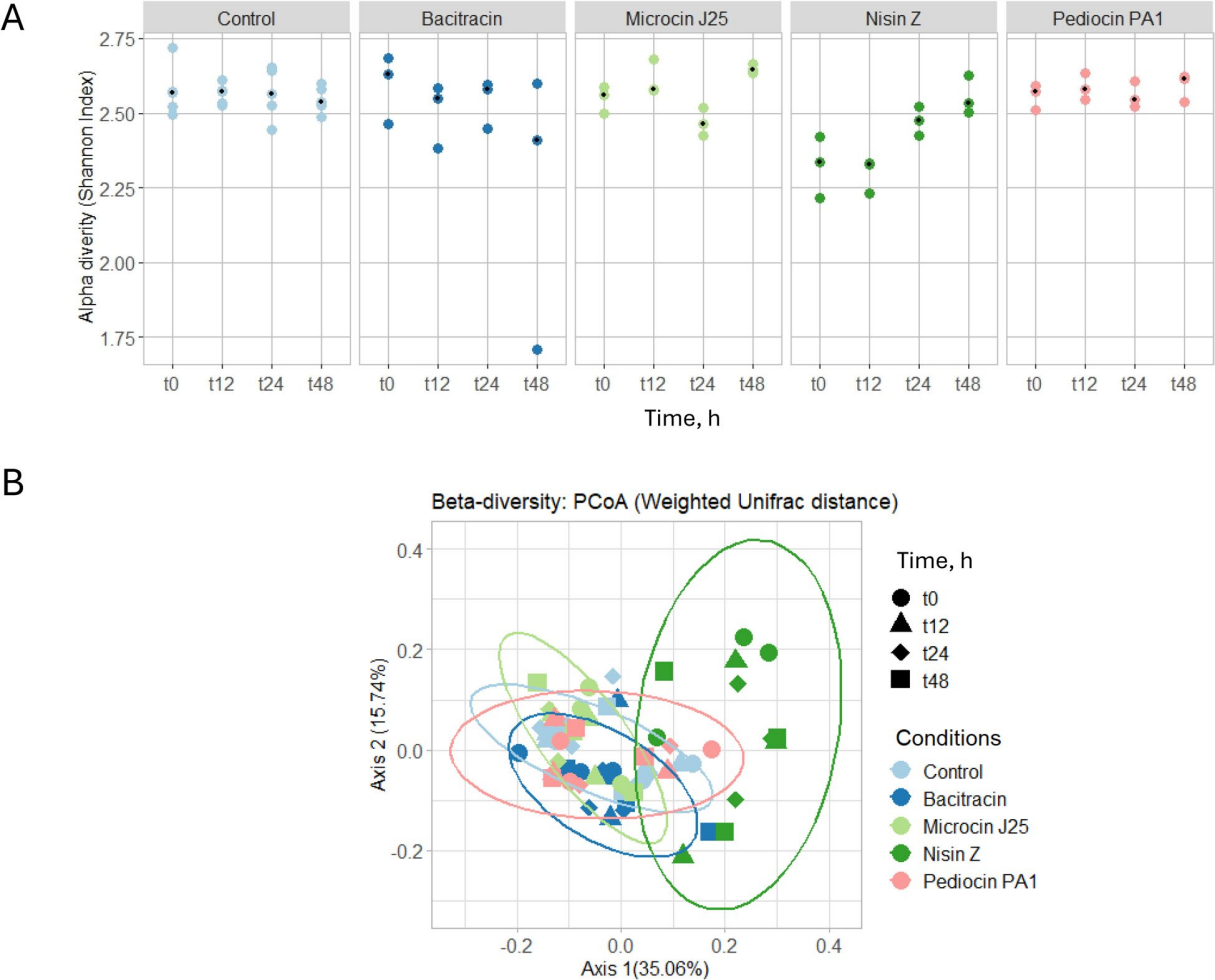
Effects of microcin J25, nisin Z and pediocin PA-1 on the diversity and richness of the colonic microbiota. **A** Alpha diversity, measured using the Shannon entropy. Kruskal–Wallis pairwise: ^*^P < 0.05. **B** Principal coordinates analysis (PCoA) of bacterial communities in the caecal fermentation based on the Weighted Unifrac distance (PERMANOVA: *P*-value = 0.002). Ellipses represent the 95% confidence interval for each treatment group. The control condition did not receive any antimicrobial agent

The alpha diversity was evaluated through the Shannon index (Fig. 4A). Alpha diversity remained stable for 48 h in the control condition where no antibacterial agents were added. Treatment with Nisin Z resulted in a significant initial decrease (at t0), followed by a gradual recovery toward the control values over time. Conversely, the addition of microcin J25 caused a significant increase in alpha diversity after 48 h. Treatment with bacitracin showed a tendency for decrease, while the use of pediocin PA-1 resulted in no observable variation in alpha diversity across the experiment duration. Beta diversity was studied using the weighted UniFrac dissimilarity matrix and visualized through Principal Coordinate Analysis (PCoA) (Fig. 4B), revealing that among the antimicrobial treatments tested, nisin Z exerted the greatest impact on the microbiota community structure.

### Effects of treatments on volatile fatty acid (VFA) metabolism

**Fig. 5:**
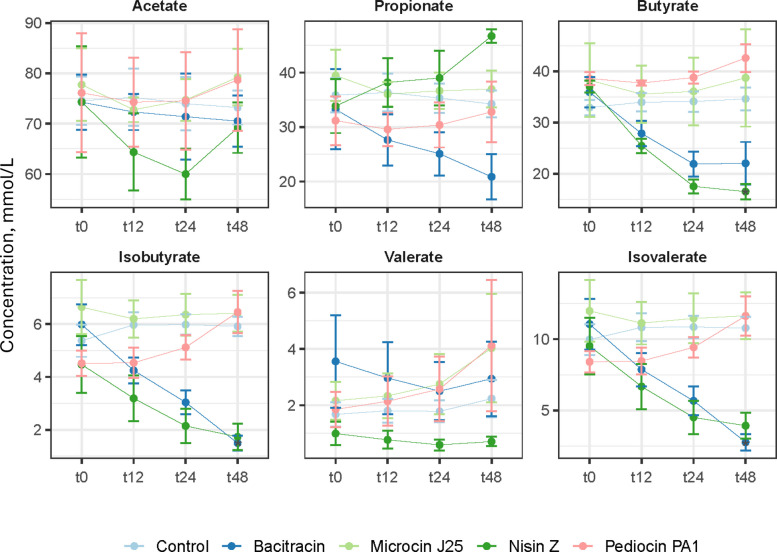
Impact of treatments on VFA concentrations. Concentration of VFAs in caecal content across treatments and time points. Statistical differences between treatments were assessed using the Kruskal–Wallis test followed by Dunn’s post hoc test with Benjamini–Hochberg (BH) correction. Significant differences are indicated for adjusted P-values < 0.05. The control condition did not receive any antimicrobial agent

The relative quantification of VFAs (Fig. 5) revealed stable levels over time in the control group, whereas bacitracin and nisin treatments significantly altered all VFA levels except for acetate, independently of time. Propionate, butyrate, isobutyrate, and isovalerate levels decreased, while valerate remained unaffected, with a significant reduction of isobutyrate and isovalerate observed at 48 h. Nisin Z induced a late increase in propionate and a significant reduction in butyrate at 48 h, as well as a decrease in isobutyrate and isovalerate production. In contrast, microcin J25 and pediocin PA-1 exhibited a more stable VFA profile.

### Effects of bacteriocins on the global caecal microbiota metabolome

**Fig. 6:**
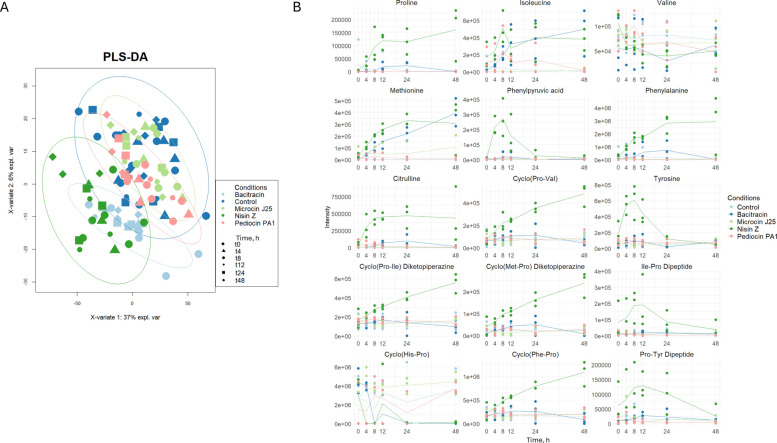
Impact of antimicrobial treatments on the caecal metabolome. **A** Partial least squares discriminant analysis (PLS-DA) score plots showing the discrimination of metabolomic profiles based on treatment groups. Each point represents a biological sample, colored by antimicrobial treatment. Ellipses represent the 95% confidence interval for each treatment group. **B** Temporal variation of intensities for selected metabolites, for each treatment. The control condition did not receive any antimicrobial agent

LC-MS was used to perform an untargeted metabolomic measurement to evaluate the impact of the selected bacteriocins on the metabolic activity of the poultry caecal microbiota*.* The LC-MS data and corresponding PCA (Fig. S4) show good consistency between replicates and between quality controls, and no clear separation between treatments. PLS-DA also showed substantial overlap between treatment-associated signatures, with only two treatments, nisin Z and bacitracin, showing a slight separation from the other ones (Fig. 6A). The VIP with a threshold superior to 2 were annotated and are listed in Table S4, revealing that nisin treatment yielded an increase in the abundance of certain amino acids (proline, methionine, phenylalanine), while others were increased only transiently between 0 and 12 h (tyrosine). The amino acid citrulline, an intermediate of microbial arginine catabolism, was also increased. A transient increase was also observed for phenylpyruvate, a microbial catabolite of phenylalanine that serves as a central intermediate in the production of bioactive aromatic metabolites. In addition, nisin Z treatment generated accumulation of several proline-containing dipeptides (Fig. 6B). The metabolites over-represented for the bacitracin condition are mainly degradation forms of bacitracin.

### Correlation between metabolites and microbial genera

**Fig. 7:**
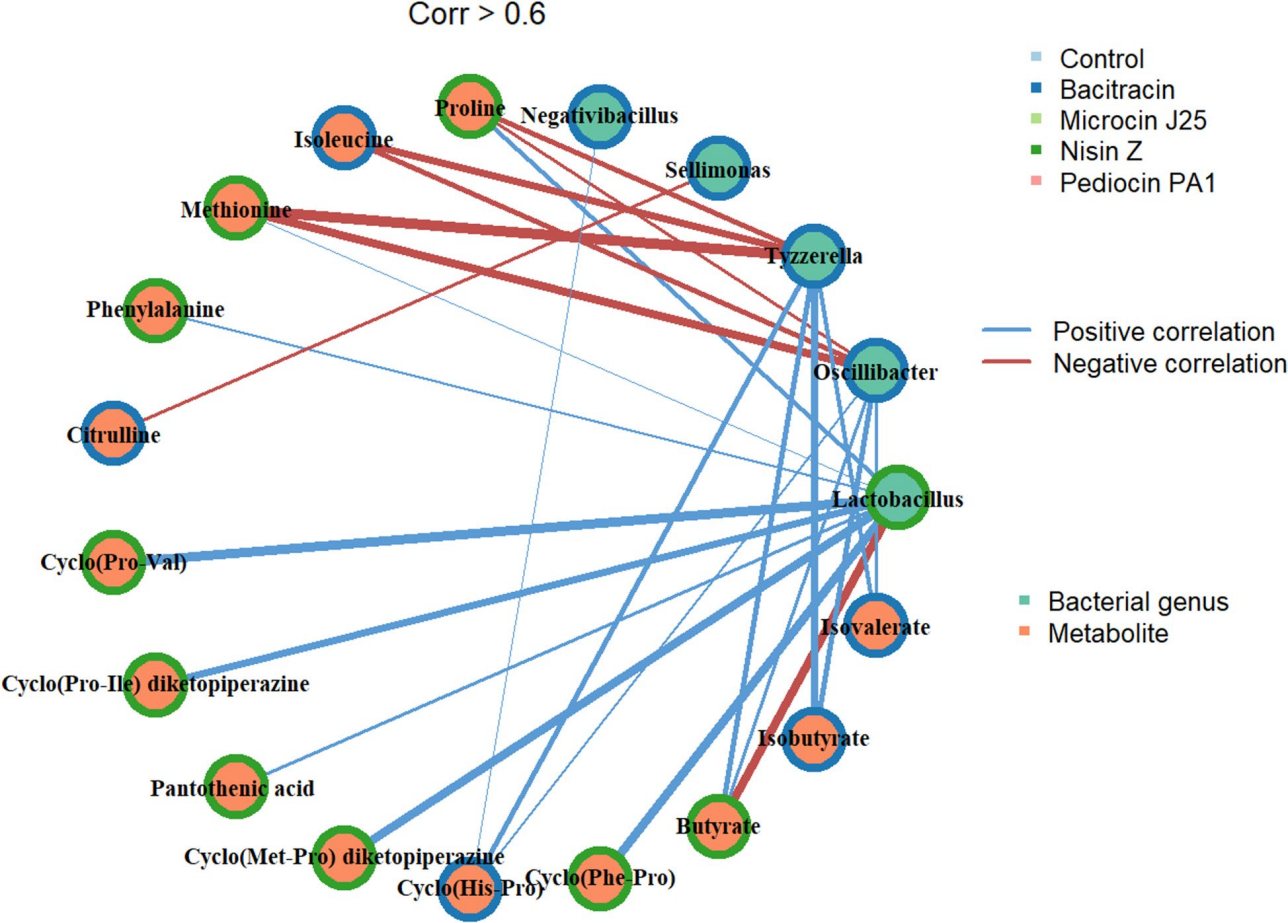
Correlation network of microbiome and metabolome. A correlation cut-off of 0.6 was applied; a red line indicates a positive correlation, and a blue line indicates a negative correlation. Edge thickness indicates the magnitude of the correlation (thicker = stronger). The colored border around each node indicates the condition in which the correlation was statistically significant. The control condition corresponds to samples that did not receive any antimicrobial agent

Correlation analysis was used to characterize the relationship network between intestinal microorganisms and metabolites (Fig. 7). Metabolites annotated as amino acids, including proline, methionine, and phenylalanine, as well as several cyclic peptides such as cyclo(Pro-Val), cyclo(Phe-Pro), cyclo(Pro-Ile) diketopiperazine, and cyclo(Met-Pro) diketopiperazine, exhibited positive correlations with *Lactobacillus*. In contrast, butyrate showed a negative correlation with *Lactobacillus* in samples from fermentations treated with nisin Z. The complete heatmap of all pairwise correlations between bacterial genera and metabolites is shown in Fig. S5.

### Predicted functional potential of the caecal microbiota

**Fig. 8:**
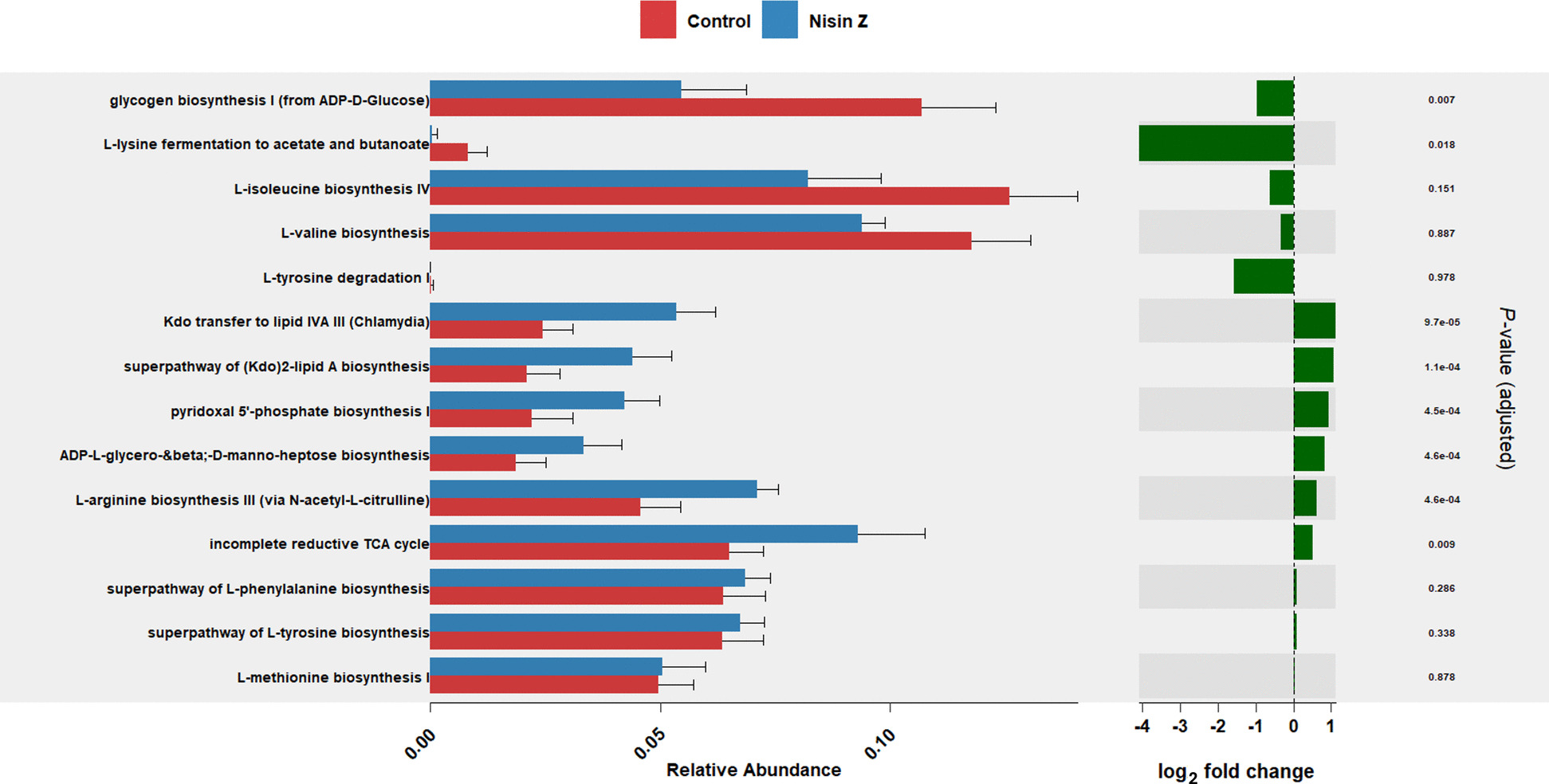
Predicted functional potential of the caecal microbiota. Bar plot showing significant MetaCyc pathways predicted by PICRUSt2. Bars indicate mean relative abundance (± SEM) for Control (red) and Nisin Z (blue). The right panel shows log fold change (green) with Benjamini–Hochberg adjusted *P*-values

To assess whether the taxonomic shifts induced by bacteriocins translated into functional changes, we performed a PICRUSt2 analysis to predict the genomic potential of the community (Fig. 8). Following nisin Z treatment, a significant reduction was observed in pathways related to primary metabolism and energy storage, including Glycogen biosynthesis (adjusted *P* = 0.032) and the TCA cycle (*P* = 0.030). Furthermore, pathways associated with survival and structural integrity, such as Vitamin B_6_ biosynthesis (*P* = 2.5e-04) and Lipid A biosynthesis (*P* = 2.6e-04), were significantly enriched in the nisin Z group compared to the control. While the predicted potential for Butanoate production showed a downward trend (log_2_ fold change < −1), the actual measured reduction in butyrate concentrations (Fig. 6) confirms a significant functional loss in the community's fermentative capacity.

### Stability and antibacterial activity of bacteriocins in poultry caecal conditions

**Fig. 9:**
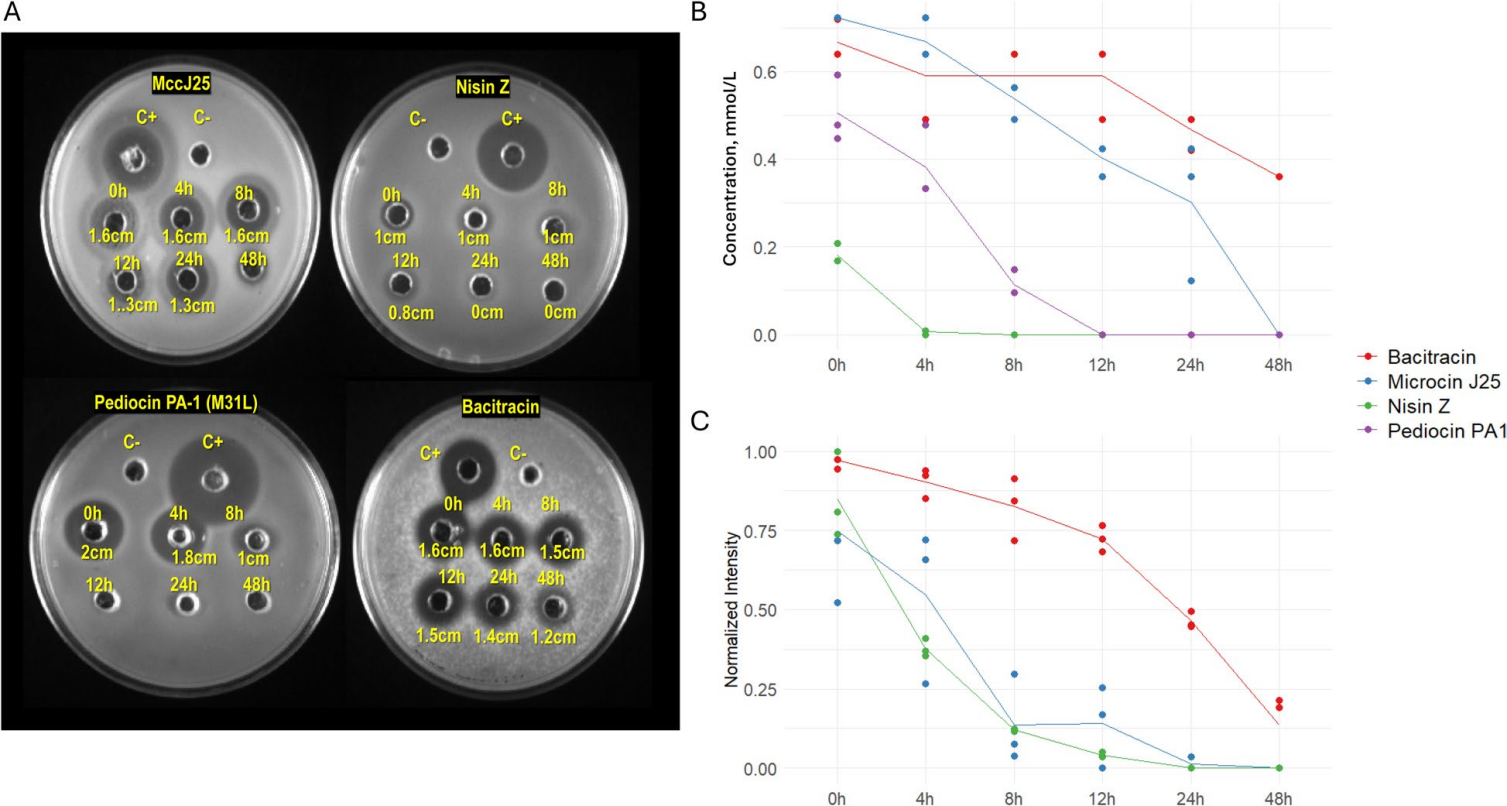
Inhibitory activity and bacteriocin quantification under poultry colonic conditions in the PolyFermS model. **A** Agar diffusion assay showing the inhibitory activity. Microcin J25 against *Salmonella enterica* subsp. *enterica* Newport ATCC 6962, nisin Z and pediocin PA-1 against *Listeria ivanovii* HPB28 and bacitracin against *Streptococcus pyogenes* RBL4 over 48 h. C+ is the positive control (microcin J25, nisin Z, pediocin PA-1 and bacitracin at 1 mg/mL); C- is the negative control (supernatant from reactor without antimicrobial). **B** Estimation of the concentration of microcin J25, nisin Z, pediocin PA-1 and bacitracin over 48 h, based on the micro-dilution method. **C** Relative abundance of microcin J25, nisin Z and bacitracin over 48 h colonic fermentation in the PolyFermS system based on extracted ion chromatogram peaks areas obtained by LC-MS

The PolyFermS system was inoculated with composite poultry microbiota obtained from the ileum and caeca of four healthy adult birds that were not given antibiotics during the rearing period. Continuous fermentation was conducted for 90 days using the experimental setup depicted in Fig. 1. The bioavailability of the bacteriocins under caecal poultry conditions and therefore in the modified VL medium was assessed by evaluating antimicrobial activity using micro-dilution and agar well diffusion assays (Fig. 9A and B). Microcin J25 and bacitracin remained active throughout 24 h of continuous fermentation. In contrast, pediocin PA-1 was effective for up to 8 h, while the inhibition of *Listeria ivanovii* by nisin Z was weak since the beginning.

Quantification to assess the stability of the three bacteriocins in the complex supernatant from the PolyFermS system was performed using both microtitration and LC-MS (Fig. 9C). The microtitration indicated that the concentration of bacitracin gradually decreased but remained relatively high even after 48 h. The concentration of microcin J25 declined more markedly than that of bacitracin, with a noticeable reduction from 12–24 h, then tended towards zero at 48 h. Nisin Z concentration fell rapidly in the first few hours, becoming virtually undetectable before 12 h. Decrease of pediocin PA-1 was rapid and similar to that of nisin Z, with almost complete disappearance before 12 h.

Using LC-MS, microcin J25, nisin Z and bacitracin could be detected and quantified until 24 h of fermentation, while pediocin PA-1 was not detected (Fig. 9C, Fig. S6 and S7).

### Analysis of microcin J25 and nisin Z degradation products

**Fig. 10:**
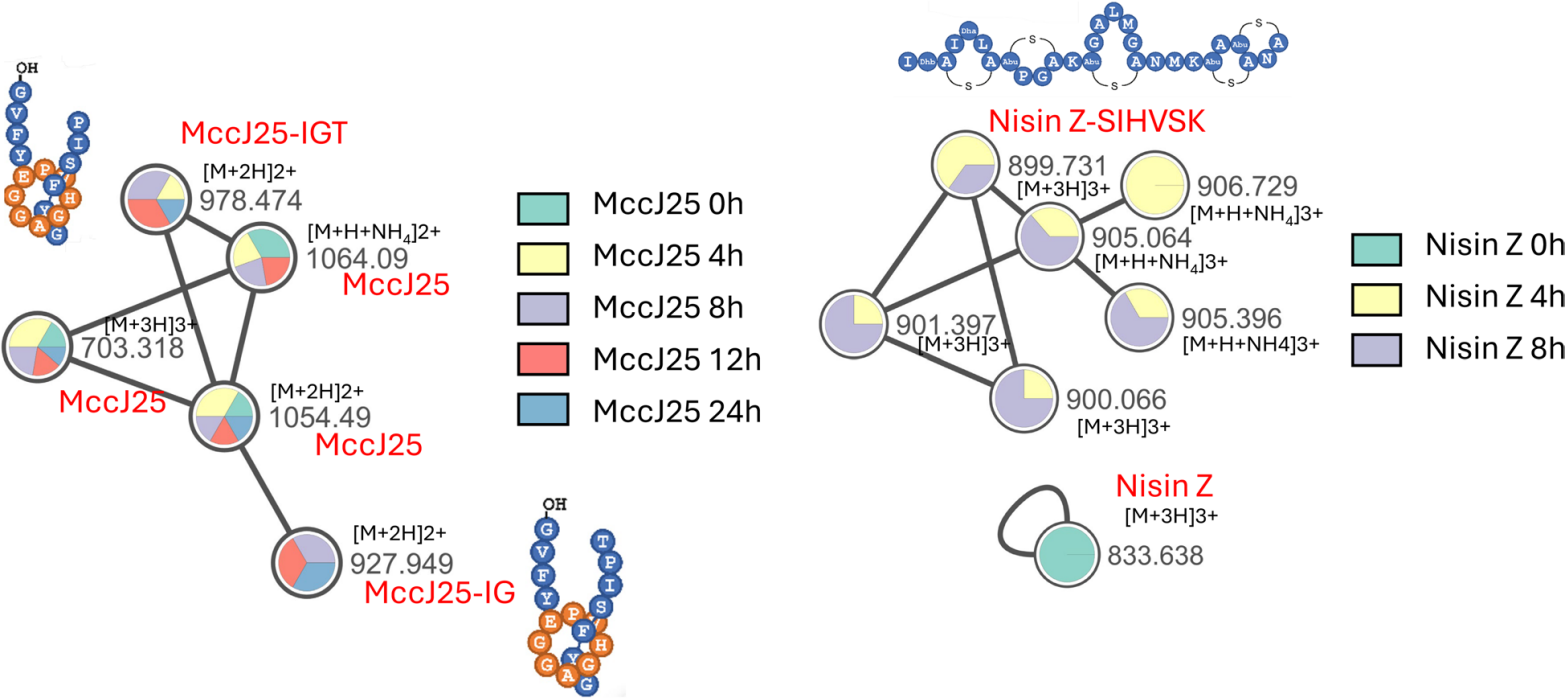
Molecular networking showing the degradation forms of bacteriocins, constructed from LC-MS/MS data of extracts at t0, t4, t8, t12, t24

Molecular networking analysis based on LC-MS/MS data from the fermented broth was used to explore the degradation products of bacteriocins (Fig. 10). The lasso and thioether-crosslinked structures of microcin J25 and nisin Z, respectively resulted in complex fragmentation patterns (Fig. S8). Microcin J25 fragmentation yielded fragment ions diagnostic of the lasso structure, as previously reported [49, 50]. These rotaxane fragments result from multiple hydrolyses in the loop region, the N and C-terminal segments being associated through the steric entrapping of the tail in the macrolactam ring. This behaviour was also observed upon hydrolysis, and several rotaxane hydrolysis products were detected over time in the PolyFermS fermentation conditions (Fig. 9). Both fragment ions at *m/z* 120.08 (Phe immonioum) and at *m/z* 659.33 (a_8_ ion corresponding to the macrolactam ring) were used as diagnostic species to detect more degradation products based on extracted ion chromatograms (Fig. S9A). This analysis revealed 6 degradation forms of microcin J25, corresponding to multiple hydrolyses in the F10-P16 region, at the top of the loop (Table 1, Fig. S10). For nisin Z, the internal fragment ion Abu23-Ala28 internal fragment ion containing 2 thioether bridges was used as a diagnostic ion (Fig. S9B). Four main degradation products were detected, resulting from cleavage of the {Ser29-Lys34} C-terminal region and oxidation of methionine residues (Table 1, Fig. S11).

**Table 1:** Antimicrobial compounds used in this study and main degradation products formed in poultry caecal conditions

Peptide	Raw formula	Mw, Da	RT, min
**Microcin J25**	**C**_**101**_**H**_**139**_**N**_**23**_**O**_**27**_	**2,106.0211**	
{Microcin J25—VGIGTP}	C_77_H_101_N_17_O_21_	1,599.73	5.6
{Microcin J25—VGIG}	C_86_H_115_N_19_O_24_	1,797.84	5.7
{Microcin J25—IGT}	C_89_H_120_N_20_O_24_	1,852.88	5.7
{Microcin J25—IG}	C_93_H_127_N_21_O_26_	1,953.93	5.8
{Microcin J25—GIG}	C_91_H_124_N_20_O_25_	1,896.90	5.9
Microcin J25 hydrolyzed	C_101_H_141_N_23_O_28_	2,124.03	6.1
**Nisin Z**	**C**_**141**_**H**_**229**_**N**_**41**_**O**_**38**_**S**_**7**_	**3,328.5292**	
Nisin Z (1–28)	C_112_H_182_N_32_O_31_S_7_	2,695.1694	6.5
Nisin Z (1–28) ox	C_112_H_182_N_32_O_32_S_7_	2,711.1643	6.3
Nisin Z (1–28) ox	C_112_H_182_N_32_O_32_S_7_	2,711.1643	6.8

Raw formula, calculated monoisotopic molecular weight (Mw) and measured retention time (RT, min) of the main ions detected

## Discussion

The widespread use of antibiotics as growth promoters has led to the emergence and dissemination of multiple resistance mechanisms among pathogenic bacteria in humans and animals. This situation underscores the urgent need for effective alternatives to antibiotics in poultry production [51]. Bacteriocins represent a promising class of antimicrobial peptides due to their distinct molecular structures and mechanisms of action, which reduce the likelihood of cross-resistance compared to conventional antibiotics [52–54]. Moreover, antibiotics often exert broad and disruptive effects on gut microbiota, causing long-term alterations that can persist for months after treatment. In contrast, bacteriocins generally display low cytotoxicity and minimal impact on microbiota composition, making them attractive candidates for targeted interventions [55]. It is therefore also necessary to study the effects of bacteriocins on the colonic microbiota over the short and long term. Antibiotics can also damage the colonic mucus barrier, leading to bacterial penetration of the mucus layer and increased risk of inflammation. This effect appears to be microbiota-independent and mediated through endoplasmic reticulum stress in colonic tissue [56]. Whereas bacteriocins were shown to have low or no cytotoxicity [57, 58].

In vivo experiments are valuable for studying the complex interactions between gut microbiota and the host, especially when evaluating feed additives or conducting challenge tests [59–61]. However, in vivo models often present significant logistical and financial challenges, along with stringent ethical regulations, which may restrict their use in large-scale screenings [62]. As a result, the use of in vitro models that can reproduce in vivo conditions is increasingly recommended. Among these, in vitro models mimicking human or animal microbiotas and their function are increasingly popular, particularly in the current context where interest is increasing in studying the biological functions of the microbiota and its links with health. While intestinal in vitro modeling systems may not fully replicate the entire host-microbe relationship, they are still very relevant for early screening stages of research including mechanistic studies. These in vitro models offer valuable insights that can guide later in vivo research and contribute to a more comprehensive understanding of gut microbial ecology and its impact on host health. Furthermore, in vitro studies are generally less expensive than in vivo counterparts and avoid the ethical concerns associated with animal experimentation [22]. Recently, we used a batch fermentation in sealed bottle model to screen the effect of various classes of bacteriocins and antibiotics at different concentrations on the chicken caecal microbiota composition and metabolic activity [23]. The batch fermentation in sealed bottle could not be maintained as the continuous fermentation PolyFermS model, where the pH and renewal of culture medium can be maintained for a long period of time.

In this study, we analyzed the impact of three bacteriocins from different classes and origins each with different mechanisms of action, on microbiota composition and metabolic activity. We also investigated the correlations between microbiota composition and the global metabolomes including VFAs, under in vitro conditions mimicking caecal conditions. Using the PolyFermS in vitro model, we succeeded in reproducing the conditions found in the poultry caecum as shown by the composition of the microbiota and predominance of Firmicutes, with Bacteroides being the second most predominant. This is in alignment with findings from other in vivo and in vitro studies using the same model [59, 63].

The differential abundance analysis chosen for our study was analysis of compositions of microbiomes with bias correction (ANCOM-BC) implemented in QIIME 2 pipeline. This method was selected because it explicitly corrects compositional and sampling biases inherent to 16S rRNA sequencing data and provides bias-adjusted log-fold change estimates with controlled false-discovery rates. Benchmarking work has shown that different DA approaches can yield highly divergent results [64], emphasizing the need for robust, composition-aware frameworks. Moreover, the choice of this method aligns with recent methodological advances [65] advocating bias-corrected multigroup models to minimize spurious associations. By prioritizing the reduction of false positives, this approach enhances the reliability of our interpretation, particularly in controlled in vitro fermentation experiments where subtle microbial shifts are expected rather than major compositional disruptions.

No significant impact of microcin J25 on the poultry caecal microbiota was observed. Similar results were obtained with swine colonic microbiota in an in vitro study using the PolyFermS [31]. In an in vivo study conducted in chickens challenged with *Escherichia* and *Salmonella*, microcin J25 was shown to increase the abundance of *Bifidobacterium* and lactic acid bacteria in fecal samples [21]. The broad impact of nisin Z on various bacterial families including Bacilli and Clostridia, aligns with the findings of Kierończyk et al. [66], who showed the effect of nisin in reducing the proliferation of *Lactobacillus* and *Eubacterium* species in the caeca. Our results also confirmed the findings of our previous batch fermentation study [23], in which we showed that, at the tested concentration, nisin Z had a more significant impact than the antibiotic.

Regarding VFA, the control condition displayed relatively stable VFA levels across all time points, suggesting that the baseline metabolic activity of the microbiota was not affected by any antimicrobial compound [67], which means that the stability of the system is observed. A significant decrease in butyrate was observed following nisin Z treatment, whereas no such effect was detected with microcin J25 or pediocin PA-1. This may be linked to the susceptibility of *Bifidobacterium*, Lachnospiraceae and Ruminococcaceae strains to nisin Z, which are involved in butyrate production [66]. Butyrate is crucial for intestinal epithelial energy, modulating immune responses and metabolism; its depletion could contribute to the emergence of diseases by promoting the growth of enteric pathogens [59, 68]. These results suggest that careful selection of bacteriocins could be used to enhance beneficial short chain fatty acid (SCFA) production in the gut, such as butyrate production, or to selectively inhibit pathogenic bacteria while minimizing disruption to the overall microbial ecosystem [69, 70].

The difference in the effects of microcin J25, nisin Z and pediocin PA-1, may be associated to their distinct mechanisms of action. Nisin Z has a dual mechanism of action, which combines via lipid II binding, the inhibition of cell wall synthesis and membrane insertion leading to pore formation, without receptor specificity. Furthermore, pediocin PA-1 also form pores, but acts through a more specific mechanism involving the Man-PTS system [19]. Microcin J25 inhibits RNA polymerase and its uptake into the cytoplasm is mediated by the receptor FhuA coupled to the TonB/ExbB/ExbD system to cross the outer membrane, and SbmA at the inner membrane [71]. This difference in the mechanisms of action leads to a different spectrum of antimicrobial activity, which may explain that nisin Z induced a more sustained impact on the microbiota, whereas microcin J25 and pediocin PA-1’s effects are more transient.

There is a limited number of metabolomics studies and data regarding the impact of bacteriocins on chicken microbiota activity. Microcin J25 did not induce any significant shifts in the metabolome, as previously demonstrated in a study on pig colon microbiota [31]. Pediocin PA-1 has a moderate impact on the global metabolome in the chicken caecal microbiota. On the other hand, alteration in microbial metabolism following the administration of nisin Z, along with the increase in amino acids and cyclic dipeptides over a 48-h period, is noteworthy due to their potential as microbiome modulators. Several microbiota strains can either act as probiotics or agents for dysbiosis via secretion of cyclic dipeptides, which can affect other microbes and their hosts [72]. Proline containing cyclic dipeptides have proved effective in disrupting biofilm formation by a variety of organisms, such as the foodborne pathogen *Listeria monocytogenes* [73], the opportunistic pathogen *Staphylococcus epidermis* [74] and the toxic shock-inducing *Staphylococcus aureus* MN8 [75]. In the presence of nisin Z, increased levels of several amino acids, including proline, leucine/isoleucine, methionine, and phenylalanine, were observed along with a decrease in butyrate production. These findings suggest that nisin Z, by altering the gut microbiota, may deplete key bacterial populations such as Lachnospiraceae and Ruminococcaceae, which are thought to be involved in the conversion of amino acids into secondary metabolites like butyrate [76, 77]. Although this relationship has yet to be demonstrated experimentally, it is supported by known metabolic pathways such as the Stickland reaction, in which one amino acid serves as an electron donor (oxidized and decarboxylated) and another one as an electron acceptor (reduced), resulting in the formation of SCFA precursors. Preferred hydrogen donors include alanine, leucine, isoleucine, valine, and histidine, while glycine, proline, ornithine, arginine, and tryptophane are common hydrogen acceptors [78]. This functional shift is quantitatively supported by our Spearman correlation data (Fig. S5) and PICRUSt2 analysis (Fig. 8), which provide direct evidence of a functional decoupling in these pathways. In our study, the accumulation of amino acids and reduction in butyrate may therefore be attributed to the depletion of microbial taxa responsible for amino acid fermentation, due to nisin Z. Further analyses, such as metatranscriptomics or metaproteomics, could provide a better understanding of these interactions and their physiological implications.

Most bacteriocins can withstand high temperatures and extreme pH [79] and are thus less labile than antibiotics. However, due to their peptidic nature, bacteriocins can be susceptible to proteases unlike conventional antibiotics. It has been shown that bacteriocins can be degraded by proteolytic enzymes such as pepsin, trypsin and chymotrypsin in the stomach or intestine [80]. Thus, they would display a lower biological half-life in nature and organic environments [81]. There are a limited number of studies regarding the stability of bacteriocins in animal or human. Measuring antimicrobial concentrations in fecal samples provides insights into drug metabolism, absorption, and excretion processes in the body [82]. Quantification of bacteriocins and their degradation products in complex matrices presents several challenges due to their low abundance and interference with other components. Various methods to address these issues were developed within the technological advance in the analytical techniques. Bottom-up proteomic approach was developed for quantitative profiling of bacteriocins in complex samples. Proteins are digested into peptides, enabling identification by database search and quantification of the original proteins [83]. An alternative to avoid the digestion of the sample and analyze it directly after extraction is the use of MS/MS-based molecular networking to reveal the degradome [50]. Here, LC-MS permitted to detect and quantify the bacteriocins microcin J25 and nisin Z, while LC-MS/MS coupled to molecular networking permitted to assess their degradomes. However, pediocin PA-1 could not be detected, although previously detected in a less complex medium [58].The higher molecular weight of pediocin PA-1 and the important matrix effect resulting from the complex fermentation medium required could explain the absence of detection of this bacteriocin.

The stability of microcin J25 and its resistance to degradation by proteases, such as those found in the GIT [48, 56], have been demonstrated in various environments and attributed to its unique lasso structure [84]. Pediocin PA-1 was observed to be stable in the stomach but completely degraded in the small intestine in an in vitro model simulating the human GIT tract [56, 85]. In the present study the bacteriocins were tested directly. Microcin J25 antibacterial activity remained potent over 24 h of continuous fermentation. For pediocin PA-1 and nisin Z the biostability, hence the bioavailability, decreased before 12 h of fermentation. For nisin Z, fragmentation of the {Ser29-Lys34} C-terminal region was previously revealed in vitro under gastro-intestinal conditions [58]. Interestingly, a nisin resistance protein was reported to be found in a non-nisin-producing *Lactobacillus lactis*. This protein proteolytically inactivates nisin by removing six amino acids from its C-terminal tail, leading to the same truncated form [86] observed on our study. *Lactobacillus* is a dominant genus in the poultry ileum [87] and also in our in vitro model. This rapid degradation of nisin Z (Fig. 9) explains the recovery of *Lactobacillus* observed at 24 h (Fig. 3), once the antimicrobial pressure was removed (Fig. 3). However, this recovery in population density did not restore the metabolic profile, as shown by the sustained depletion of butyrate and accumulation of amino acids (Fig. 6). Thus, the cleavage observed likely result from the secretion of protease involved in nisin inactivation by *Lactobacillus* strains. However, this degradation remains controversial, as it was previously proved that in vitro nisin completely degraded following small intestinal digestion [88] while O’Reilly et al. [17] showed that orally ingested non-encapsulated nisin could reach the lower gastrointestinal tract (GIT) intact in a swine model.

When bacteriocins are shown to be stable in the GIT, it is essential to demonstrate their non-toxicity to various cellular systems within this environment. Data from previous studies by our group indicate that these bacteriocins are non-toxic to eukaryotic cell lines [58]. However, further in vivo studies are necessary to confirm these findings and to evaluate the effects of long-term exposure to bacteriocins in the poultry model. In medical and veterinary contexts, the method of bacteriocin administration is influenced by its ability to withstand various gastrointestinal barriers, including low stomach pH and the presence of numerous proteolytic enzymes in the small intestine [85]. Given that most bacteriocins primarily target the colon, less stable bacteriocins in the GIT need protection from digestive enzymes, leading to the development of bioengineering and encapsulation technologies to overcome such challenges [89–93]. Bacteriocins can be effectively used as probiotics or live biotherapeutics, delivering doses directly to the target site [94, 95]. Bacteriocin-producing strains closely related to target pathogens may offer advantages by occupying the same ecological niche, potentially enhancing colonization resistance [96]. However, the plasmid-encoded nature of many bacteriocins raises concerns, as producers can conjugate with native enterococci, potentially aiding the survival of multidrug-resistant strains. Thus, while promising, the development of bacteriocin producers as targeted live biotherapeutics requires careful consideration of such risks [13].

## Conclusion

This study demonstrates that bacteriocins exert distinct, concentration-dependent effects on poultry caecal microbiota and metabolic activity within a controlled PolyFermS fermentation model. While nisin Z significantly altered microbial composition and the metabolome, microcin J25 and pediocin PA-1 showed a higher degree of selectivity, with minimal impact on microbiota stability under these conditions. These preliminary results suggest that microcin J25 and pediocin PA-1 are candidates for antibiotic alternatives, though their efficacy remains to be established in a complex living system. Future research should focus on in vivo trials in broiler chickens to address the inherent limitations of in vitro models, specifically regarding bacteriocin stability and bioavailability within the dynamic gastrointestinal environment.

## Supplementary Information

Additional file 1: Table S1. Minimum inhibitory concentrations (MIC) of the antimicrobial compounds tested. Table S2. Composition of modified Viande Levure medium. Table S3. Primers for detection of specific bacterial groups by PMA-qPCR method and library sequency 16S rRNA gene. Table S4. Annotation of VIP superior to 2 from PLS-DA analysis. Fig. S1. Bacteriocin purification quality control. LC profiles (total ion chromatograms on the left) and MS spectra (on the right) of (A) MccJ25, (B) nisin Z and (C) pediocin PA-1 (M31L). Fig. S2. Microbial composition in the caecal inoculum and reactor after initial stabilization of 20 d as determined by PMA-qPCR. Fig. S3. Evolution of volatile fatty acid (VFA) composition in the inoculated reactor (IR) throughout the experimental period. Relative abundance (%) of individual VFAs (Acetic, Propionic, Isobutyric, Butyric, Isovaleric, Valeric, and Hexanoic acids) is shown as a function of time (d). Fig. S4. Impact of treatments on the caecal microbiota metabolome. Fig. S5. Heatmap of significant Spearman correlations between bacterial genera and metabolites. Fig. S6. LC-MS profiles of the caecal content extracts and detection of the antibacterial compounds introduced at t0. Fig. S7. LC-MS detection of MccJ25 (MccJ25), nisin Z and bacitracin during the caecal fermentation in the PolyFermS system. Fig. S8. MS/MS spectra of the [M+2H]^2+^ species of MccJ25 and the [M+4H]^4+^ species of nisin Z. Fig. S9. Search of degradation products of MccJ25 and nisin Z using extracted ions chromatograms (EIC) of diagnostic fragment ions. Fig. S10. MS/MS spectra of the main degradation products of MccJ25 at the [M+2H]^2+^. Fig. S11. MS/MS spectra of the main degradation products of nisin Z at the [M+3H]^3+^. 

## Data Availability

The fermentation bacterial sequences supporting the findings of this study are available at the National Center for Biotechnology Information Sequence Read Archive (NCBI SRA) under the BioProject ID PRJNA1321949.

## Abbreviations

ANCOM-BC: Analysis of compositions of microbiomes with bias correction
CCAC: Canadian Council on Animal Care
CFU: Colony-forming unit
CID: Collision-induced dissociation
ddPCR: Droplet digital polymerase chain reaction
ESI-Q-TOF: Electrospray ionization-quadrupole-time of flight
FDR: False discovery rate
GC-FID: Gas chromatography coupled with flame ionization detector
GIT: Gastrointestinal tract
GNPS: Global Natural Products Social Molecular Networking
HPLC: High-performance liquid chromatography
IBIS: Institute for Integrative Systems Biology (Institut de Biologie Intégrative et des Systèmes)
IR: Inoculum reactor
LC-MS: Liquid chromatography coupled with mass spectrometry
LC-MS/MS: Liquid chromatography-tandem mass spectrometry
LC-HRMS: Liquid chromatography-high-resolution mass spectrometry
Man-PTS: Mannose phosphotransferase system
MIC: Minimum inhibitory concentration
NC: Negative control
PCA: Principal component analysis
PCoA: Principal coordinate analysis
PERMANOVA: Permutational multivariate analysis of variance
PLS-DA: Partial least squares discriminant analysis
PMA-qPCR: Propidium monoazide-coupled quantitative polymerase chain reaction
RP-HPLC: Reverse-phase high-performance liquid chromatography
rRNA: Ribosomal ribonucleic acid
SCFA: Short-chain fatty acid
SPE: Solid-phase extraction
SPPS: Solid-phase peptide synthesis
TR: Test reactor
UPLC: Ultra-performance liquid chromatography
VFA: Volatile fatty acid
VIP: Variable importance in projection
VL: Viande Levure
