# Supplementary material for: Uncovering how selected potent bacteriocins reshape the broiler chicken gut microbiome in a PolyFermS continuous in vitro model
Source: J Anim Sci Biotechnol. 2026 Jun 12;17:117. doi: 10.1186/s40104-026-01431-w (PMC13262205; doi:10.1186/s40104-026-01431-w)
Supplement: Supplementary file 1 — Additional file 1: Table S1. Minimum inhibitory concentrations (MIC) of the antimicrobial compounds tested. Table S2. Composition of modified Viande Levure medium. Table S3. Primers for detection of specific bacterial groups by PMA-qPCR method and library sequency 16S rRNA gene. Table S4. Annotation of VIP superior to 2 from PLS-DA analysis. Fig. S1. Bacteriocin purification quality control. LC profiles (total ion chromatograms on the left) and MS spectra (on the right) of (A) MccJ25, (B) nisin Z and (C) pediocin PA-1 (M31L). Fig. S2. Microbial composition in the caecal inoculum and reactor after initial stabilization of 20 d as determined by PMA-qPCR. Fig. S3. Evolution of volatile fatty acid (VFA) composition in the inoculated reactor (IR) throughout the experimental period. Relative abundance (%) of individual VFAs (Acetic, Propionic, Isobutyric, Butyric, Isovaleric, Valeric, and Hexanoic acids) is shown as a function of time (d). Fig. S4. Impact of treatments on the caecal microbiota metabolome. Fig. S5. Heatmap of significant Spearman correlations between bacterial genera and metabolites. Fig. S6. LC-MS profiles of the caecal content extracts and detection of the antibacterial compounds introduced at t0. Fig. S7. LC-MS detection of MccJ25 (MccJ25), nisin Z and bacitracin during the caecal fermentation in the PolyFermS system. Fig. S8. MS/MS spectra of the [M+2H]2+ species of MccJ25 and the [M+4H]4+ species of nisin Z. Fig. S9. Search of degradation products of MccJ25 and nisin Z using extracted ions chromatograms (EIC) of diagnostic fragment ions. Fig. S10. MS/MS spectra of the main degradation products of MccJ25 at the [M+2H]2+. Fig. S11. MS/MS spectra of the main degradation products of nisin Z at the [M+3H]3+. [file 40104_2026_1431_MOESM1_ESM.docx]

**Supplementary material**

**Table S1.** Minimum inhibitory concentrations (MIC) of the antimicrobial compounds tested.

| **Antimicrobial compound** | **MIC (µM)** | **Sensitive strain** | **Reference** |
| --- | --- | --- | --- |
| Bacitracin | 0.17 | *Clostridium perfringens CP4* | (Charlebois et al.. 2014) |
| MccJ25 | 0.03 | *Salmonella enterica* subsp. *enterica* Newport ATCC 6962 | (Telhig et al.. 2022) |
| Nisin Z | 0.18 | *Listeria ivanovii HPB28* | (Soltani et al.. 2022) |
| Pediocin PA-1 (M31L) | 0.12 | *Listeria ivanovii HPB28* | (Soltani et al.. 2022) |

**Table S2.** Composition of modified Viande Levure medium

| **Constituent** | **Amount** |
| --- | --- |
| Fructooligosaccharides (FOS) | 2.5 g/L |
| Pectin (Citrus) | 2.5 g/L |
| Beef extract | 2.4 g/L |
| Yeast extract | 5.0 g/L |
| Maltodextrin | 2.5 g/L |
| Tryptose | 10.0 g/L |
| L-cysteine hydrochloride | 0.8 g/L |
| NaCl | 5.0 g/L |
| Mucin | 2.0 g/L |
| Uric acid | 0.7 g/L |
| Tween 80 | 1 mL |
| Bile salts | 0.4 g/L |
| KH_2_PO_4_ | 0.5 g/L |
| NaHCO_3_ | 1.5 g/L |
| KCl | 4.5 g/L |
| MgSO_4_ anhydrous | 0.6 g/L |
| CaCl_2_ x 2H_2_O | 0.1 g/L |
| MnCl_2_ x 4H_2_O | 0.2 g/L |
| FeSO_4_ x 7H_2_O | 0.005 g/L |
| Hemin solution* | 0.05 g/L |
| Vitamin solution** | 1 mL |
|  |  |

*Hemin was prepared by dissolving 1% (v/v) in ethanol to ensure complete solubilization, then diluted with distilled water and sterilized by filtration.

**The vitamin mix contained the following components per liter: 20 mg pyridoxine HCl (B6), 10 mg 4-aminobenzoic acid (PABA), 10 mg nicotinic acid (B3), 4 mg biotin (H), 4 mg folic acid (B9), 1 mg cyanocobalamin (B12), 10 mg thiamine (B1), 10 mg riboflavin (B2), 2 mg menadione (K3), 0.015 mg phyloquinone (K1), and 20 mg pantothenate (B5).

| Primer | Sequence 5’ – 3’ | Target | Reference |
| --- | --- | --- | --- |
| Eub338F  Eub518R | ACTCCTACGGGAGGCAGCAGATTACCGCGGCTVGCTGG | Total bacteria | Guo *et al.* (2008) |
| Firm 934F  Firm 1060R | GGAGYATGTGGTTTAATTCGAAGCAAGCTGACGACAACCA TGCAC | Firmicutes | Guo *et al.* (2008) |
| Bac303F  Bfr-Femrev | GAAGGTCCCCCACATTGCGCKACTTGGCTGGTTCAG | Bacteroidetes | Ramirez-Farias *et al.* (2009) |
| RumiF  RumiR | ACTGAGAGGTTGAACGGCCACCTTTACACCCAGTAAWTCCGGA | *Ruminococcaceae* | Garcia-Mazcorro *et al*. (2012) |
| F_Lacto 05  R_Lacto 04 | AGCAGTAGGGAATCTTCCACGCCACTGGTGTTCYTCCATATA | *Lactobacillaceae*. | Furet *et al.* (2009) |
| Bifi_F  Bifi_R | TCGCGTCYGGTGTGAAAGCCACATCCAGCRTCCAC | *Bidfidobacteriaceae* | Meimandipour *et al.* (2010) |
| Eco1457F  Eco1652R | CATTGACGTTACCCGCAGAAGAAGCCTCTACGAGACTCAAGCTTGC | *Enterobacteriaceae* | Bartosch *et al*. (2004) |
| 341F  805R | TCGTCGGCAGCGTCAGATGTGTATAAGAGACAGCTACGGGNGGCWGCAG  GTCTCGTGGGCTCGGAGATGTGTATAAGAGACAGGACTACHVGGGTATCTAATCC | V3-V4 hypervariable region of the 16 S rRNA gene |  |

**Table S3**. Primers for detection of specific bacterial groups by PMA-qPCR method and library sequency 16S rRNA gene

**Table S4.** Annotation of VIP superior to 2 from PLS-DA analysis

| m/z | RT (sec) | Adduct | Raw formula | Compound Name |
| --- | --- | --- | --- | --- |
| 116.071 | 62.266 | [M+H]^+^ | C_5_H_9_NO_2_ | Pro |
| 132.102 | 87.095 | [M+H]^+^ | C_6_H_13_NO_2_ | Ile |
| 140.068 | 51.679 | [M+2H]^2+^ | C_5_H_11_NO_2_ | Val |
| 150.058 | 73.405 | [M+H]^+^ | C_5_H_11_NO_2_S | Met |
| 163.060 | 59.720 | [M+H]^+^ | C₉H₈O₃ | Phenylpyruvic acid |
| 166.086 | 181.523 | [M+H]^+^ | C_9_H_11_NO_2_ | Phenylalanine |
| 176.10 | 57.230 | [M+H]^+^ | C_6_H_13_N_3_O_3_ | Citrulline |
| 197.129 | 321.839 | [M+H]^+^ | C_10_H_16_N_2_O_2_ | Cyclo(Pro Val) |
| 204.087 | 62.778 | [M+H-H_2_O]^+^ | C₉H₁₁NO₃ | Tyr |
| 211.145 | 403.527 | [M+H]^+^ | C_11_H_18_N_2_O_2_ | Cyclo Pro-Ile  diketopiperazine |
| 229.101 | 357.609 | [M+H]^+^ | C_10_H_16_N_2_O_2_S | Cyclo Met-Pro diketopiperazine |
| 230.16 | 216.56 | [M+H]^+^ | C_11_H_20_N_2_O_3_ | Ile-Pro dipeptide |
| 230.157 | 216.558 | [M+H]^+^ | C_14_H_16_N_2_O_2_ | Cyclo(Phe-Pro) |
| 235.119 | 63.210 | [M+H]^+^ | C₁₁H₁₆N₄O₂ | Cyclo(His-Pro) |
| 279.133 | 225.821 | [M+H]^+^ | C_14_H_18_N_2_O_4_ | Pro-Tyr dipeptide |


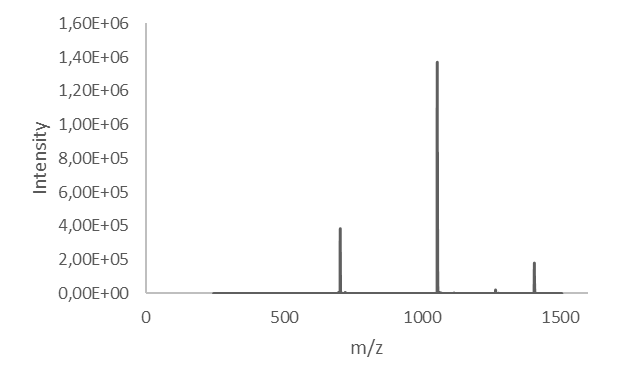

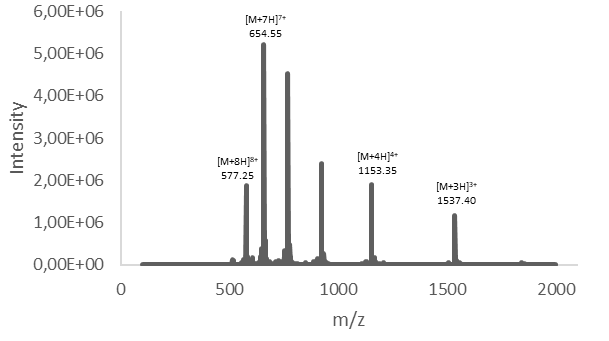

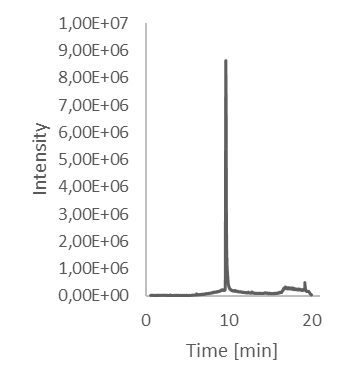


B

[M+2H]^2+^

1054,53

[M+3H]^3+^

703,35

A


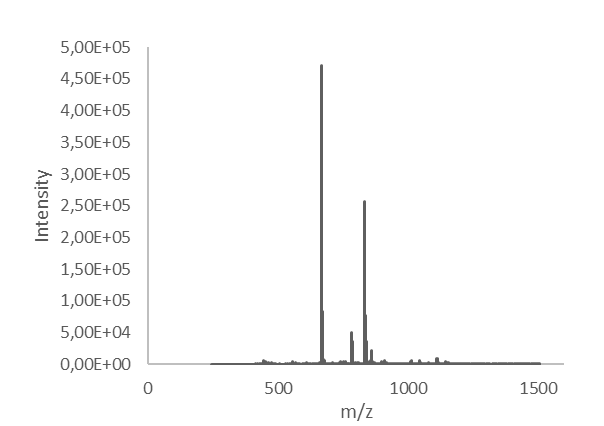

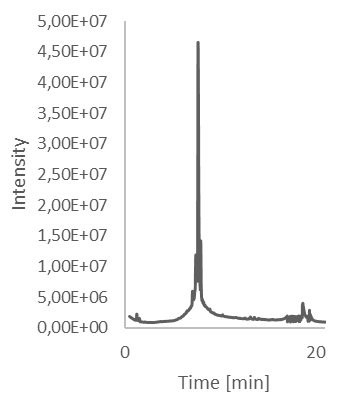

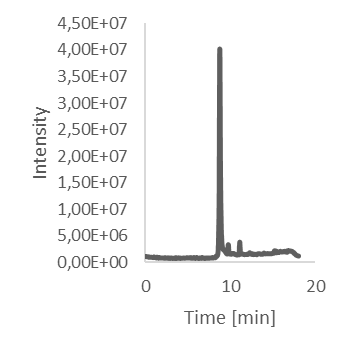


C

[M+5H]^5+^

922.90

[M+6H]^6+^

769.30

[M+5H]^5+^

667.20

[M+4H]^4+^

833.65

[M+3H]^3+^

1111.15

**Figure S1.** Bacteriocin purification quality control. LC profiles (total ion chromatograms on the left) and MS spectra (on the right) of (A) MccJ25, (B) nisin Z and (C) pediocin PA-1 (M31L).


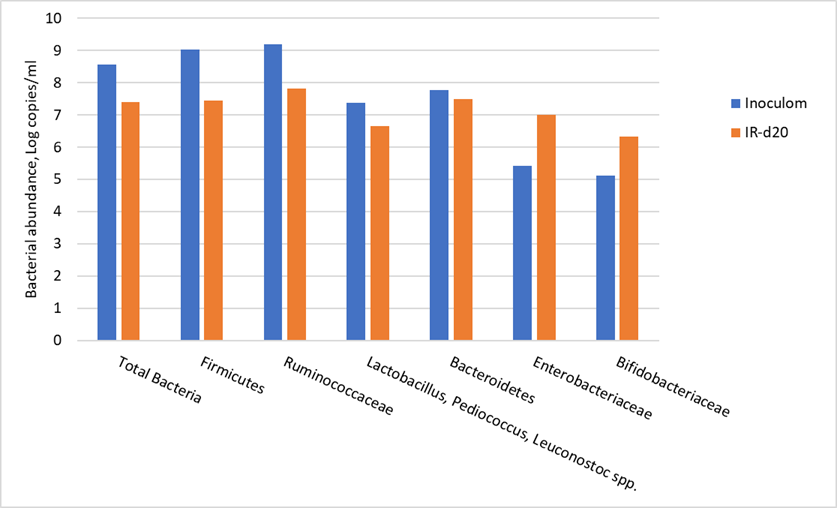


**Figure S2.** Microbial composition in the caecal inoculum and reactor after initial stabilisation of 20 days as determined by PMA-qPCR.


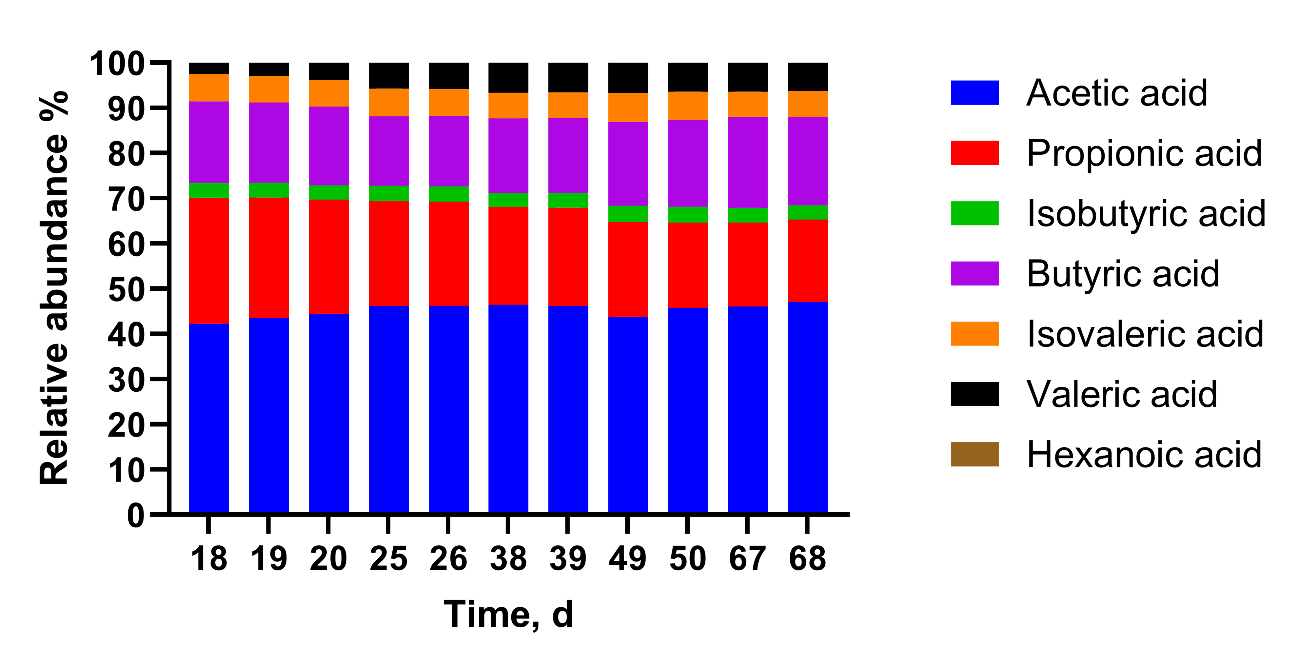


**Figure S3.** Evolution of Volatile Fatty Acid (VFA) composition in the Inoculated Reactor (IR) throughout the experimental period. Relative abundance (%) of individual VFAs (Acetic, Propionic, Isobutyric, Butyric, Isovaleric, Valeric, and Hexanoic acids) is shown as a function of time (days).

| A | 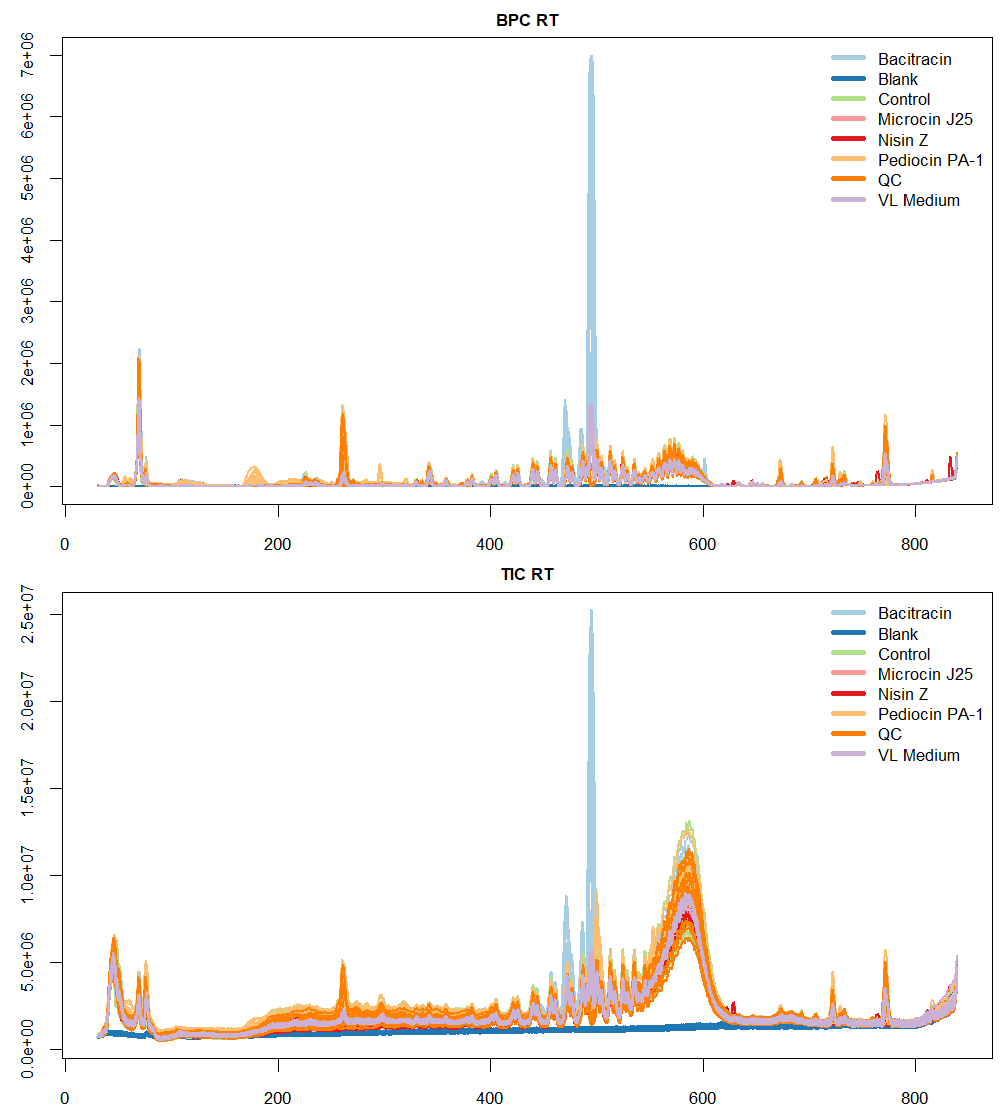 |
| --- | --- |
| B | 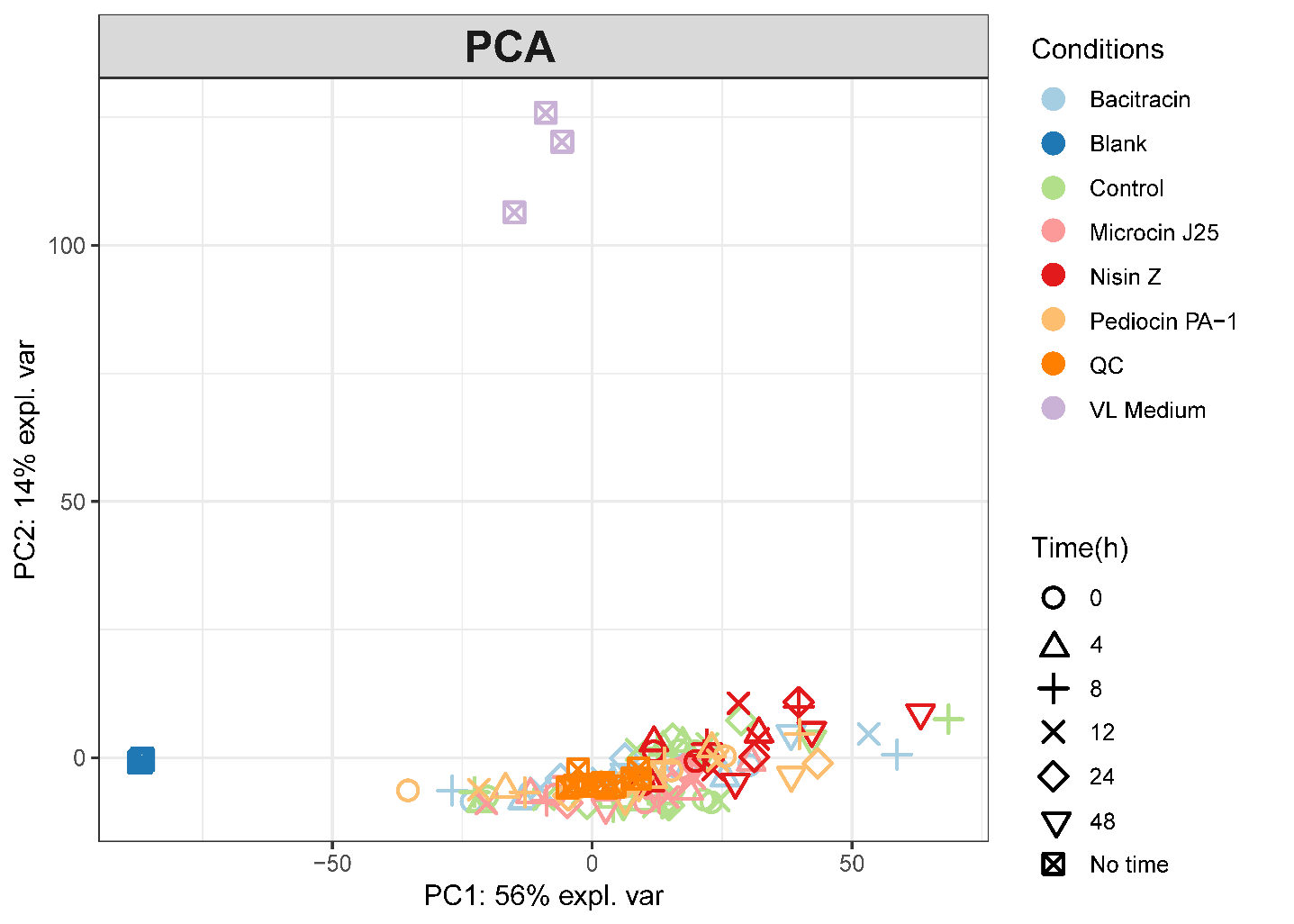 |

**Figure S4.** Impact of treatments on the caecal microbiota metabolome. (A) Base Peak Chromatograms (BPC) and Total Ion Chromatograms (TIC) collected for every sample, colored per treatment. QC corresponds to the quality control. (B) PCA of the multivariate matrix generated from LC-MS data. Blank corresponds to the extraction solvent; QC represents a pooled mixture of all samples; the control condition did not receive any antimicrobial agent.


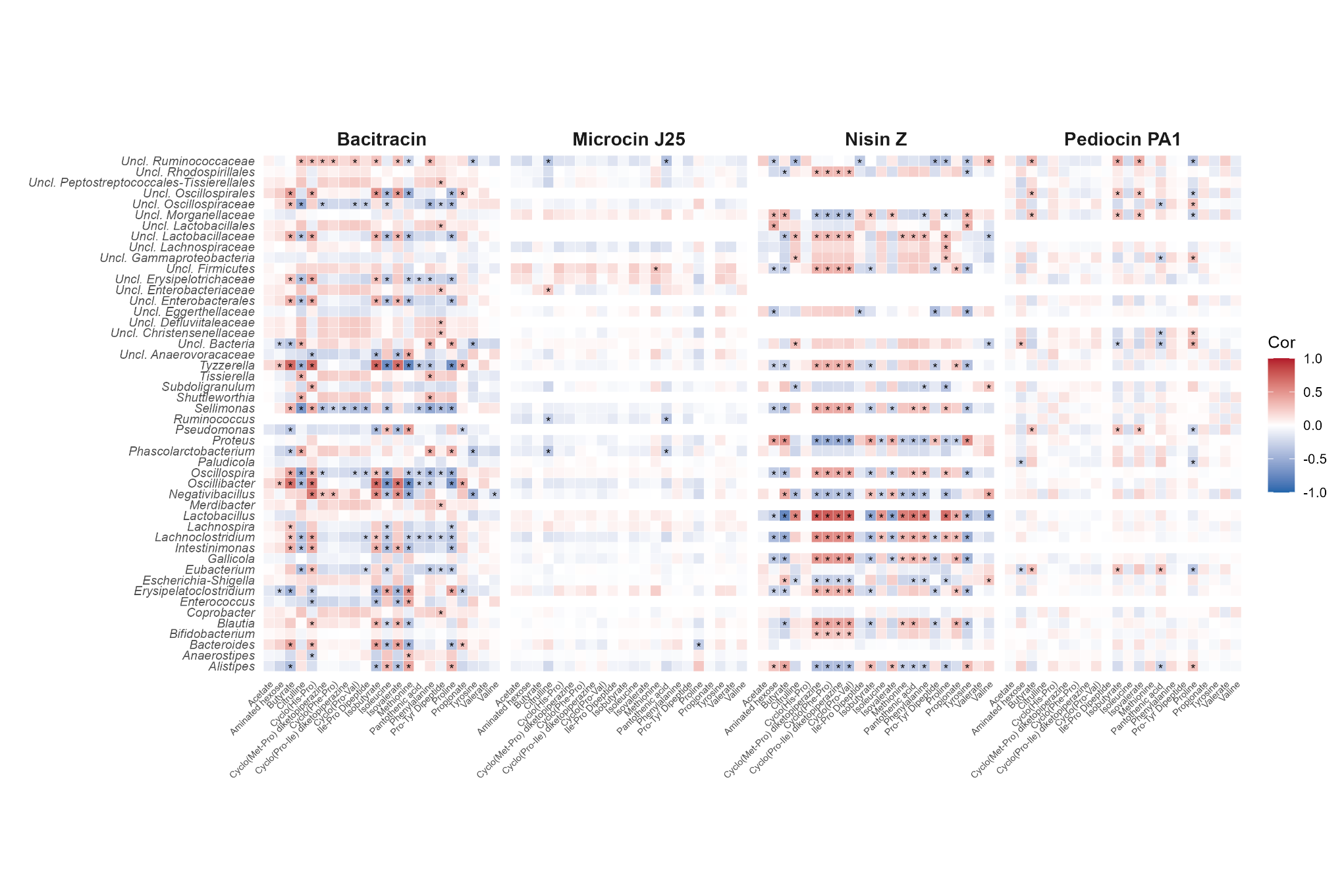
**Figure S5.** Heatmap of significant Spearman correlations between bacterial genera and metabolites. Red squares indicate positive correlations, and blue squares indicate negative correlations, non-significant correlations in white. Significance was determined using Spearman correlation with Benjamini–Hochberg correction (adjusted *p* < 0.05).

**A**

**B**

**C**

**D**

Polymer contamination

**Figure S6.** LC-MS profiles of the caecal content extracts and detection of the antibacterial compounds introduced at t0. (A) extracted ion chromatogram of the [M+3H]^3+^ species of bacitracin (*m/z* 474.92) in a bacitracin-treated sample, (B) extracted ion chromatogram of the [M+3H]^3+^ species of MccJ25 (*m/z* 703.01) in a MccJ25-treated sample, (C) extracted ion chromatogram of the [M+5H]^5+^ species of nisin Z (*m/z* 666.71) in a nisin Z-treated sample, (D) extracted ion chromatogram of the [M+5H]^5+^ species of pediocin PA-1 (M31L) (*m/z* 921.62) in a pediocin PA-1 (M31L)-treated sample.


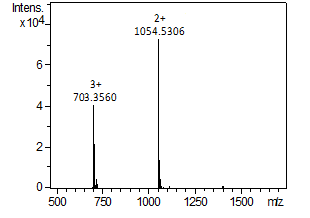

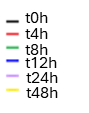

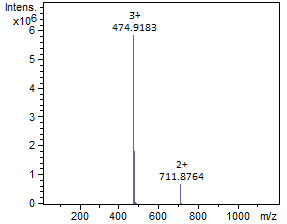


**C**

**D**

**A**

**B**


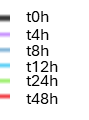


**E**

**F**


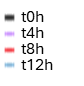

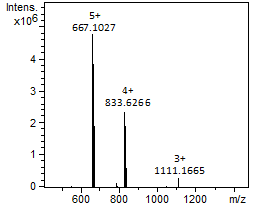


**Figure S7.** LC-MS detection of MccJ25 (MccJ25), nisin Z and bacitracin during the caecal fermentation in the PolyfermS system. (A) Extracted ion chromatogram of the [M+3H]^3+^ ion of bacitracin (*m/z* 474.0); (B) Mass spectrum of bacitracin; (C) Extracted ion chromatogram of the [M+3H]^3+^ ion of MccJ25 (*m/z* 703.0); (D) Mass spectrum of MccJ25; (E) Extracted ion chromatogram of the [M+5H]^5+^ ion of nisin Z (*m/z* 667.0); (f) Mass spectrum of nisin Z.

| A | 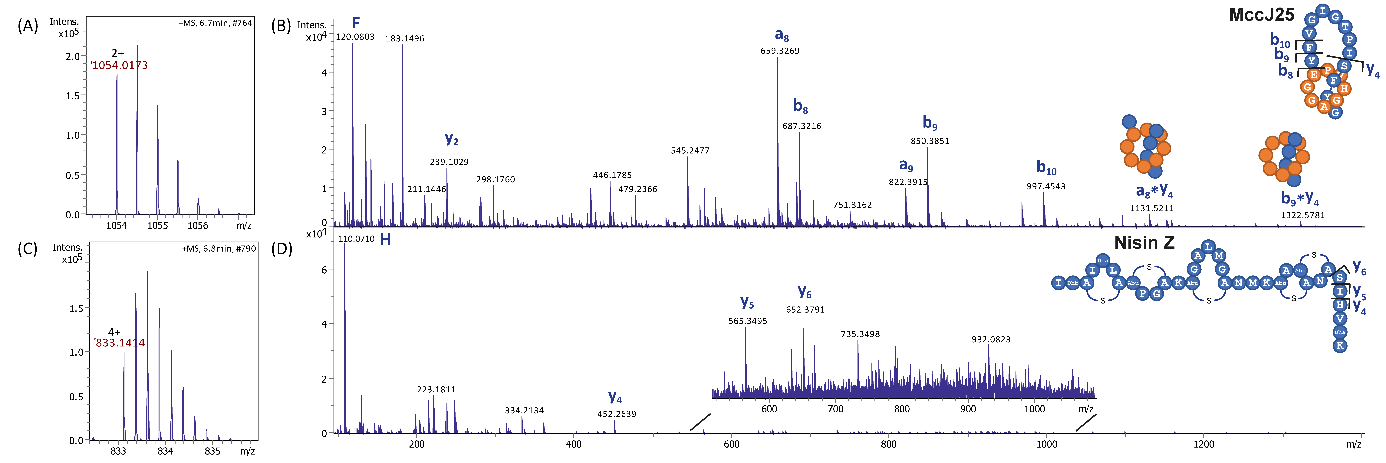 |
| --- | --- |
| B |  |

**Figure S8.** MS/MS spectra of (A) the [M+2H]^2+^ species of MccJ25 (*m/z* 1054.02) and (B) the [M+4H]^4+^ species of nisin Z (*m/z* 833.14). The rotaxane fragment ions of MccJ25, where b_i_ and y_j_ ions remain non-covalently associated, are noted b_i_ * y_j_.


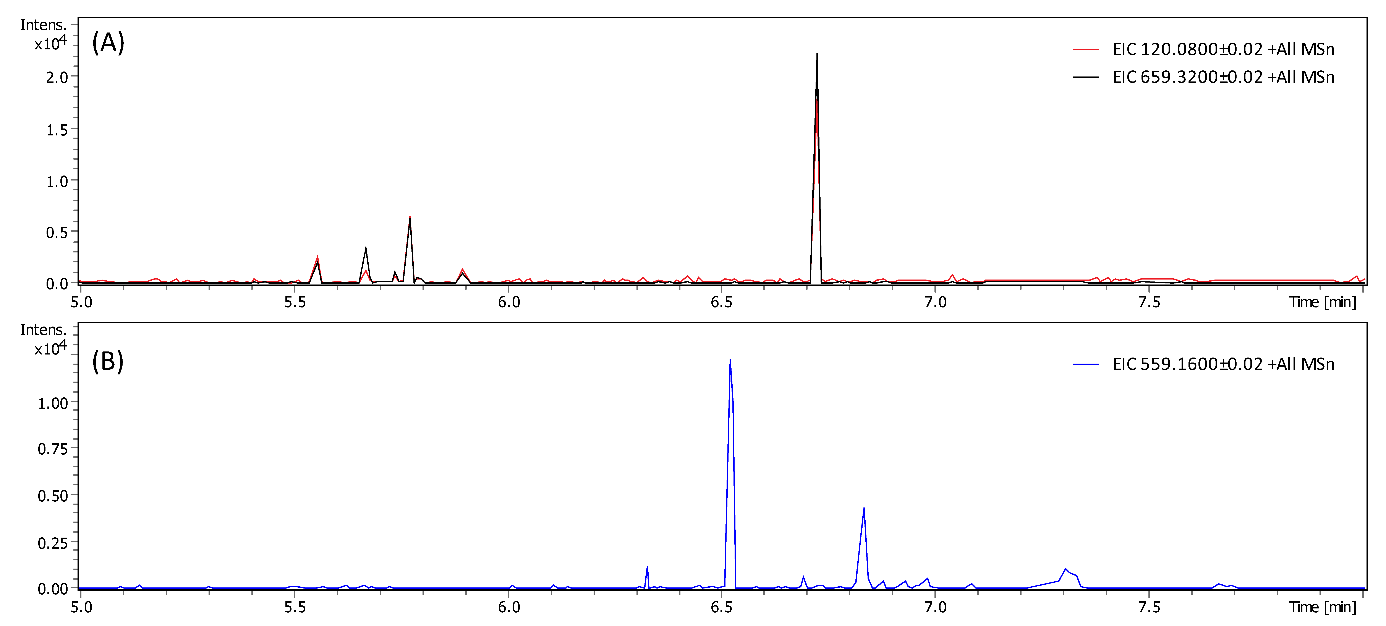


**Figure S9.** Search of degradation products of (A) MccJ25 and (B) nisin Z using extracted ions chromatograms (EIC) of diagnostic fragment ions (at *m/z* 120.08 and *m/z* 659.32 corresponding to Phe immonium and a_8_, respectively for MccJ25 and at *m/z* 559.16 corresponding to the Abu23-Ala28 internal fragment ion containing 2 thioether bridges for nisin Z).

| A | 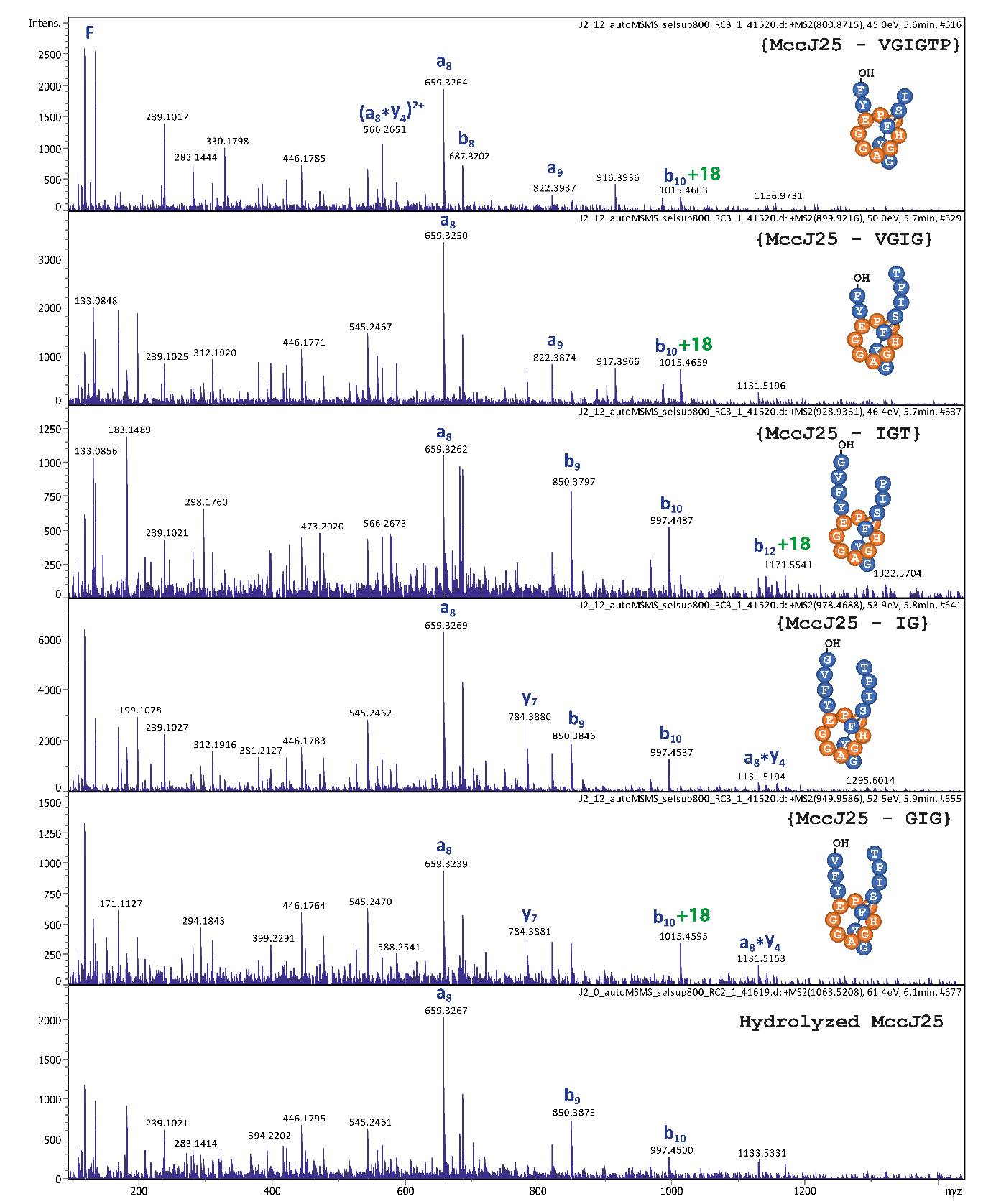 |
| --- | --- |
| B |  |
| C |  |
| D |  |
| E |  |
| F |  |

**Figure S10.** MS/MS spectra of the main degradation products of MccJ25 at the [M+2H]^2+^.(A) MccJ25 – VGIGTP (*m/z* 800.87); (B) MccJ25 – VGIG (*m/z* 899.92); (C) MccJ25 – IGT (*m/z* 927.4465); (D) MccJ25 – IG(*m/z* 977.9703); MccJ25 – GIG (*m/z* 949.4596) and MccJ25 hydrolyzed (*m/z* 1063.0255).

| A | 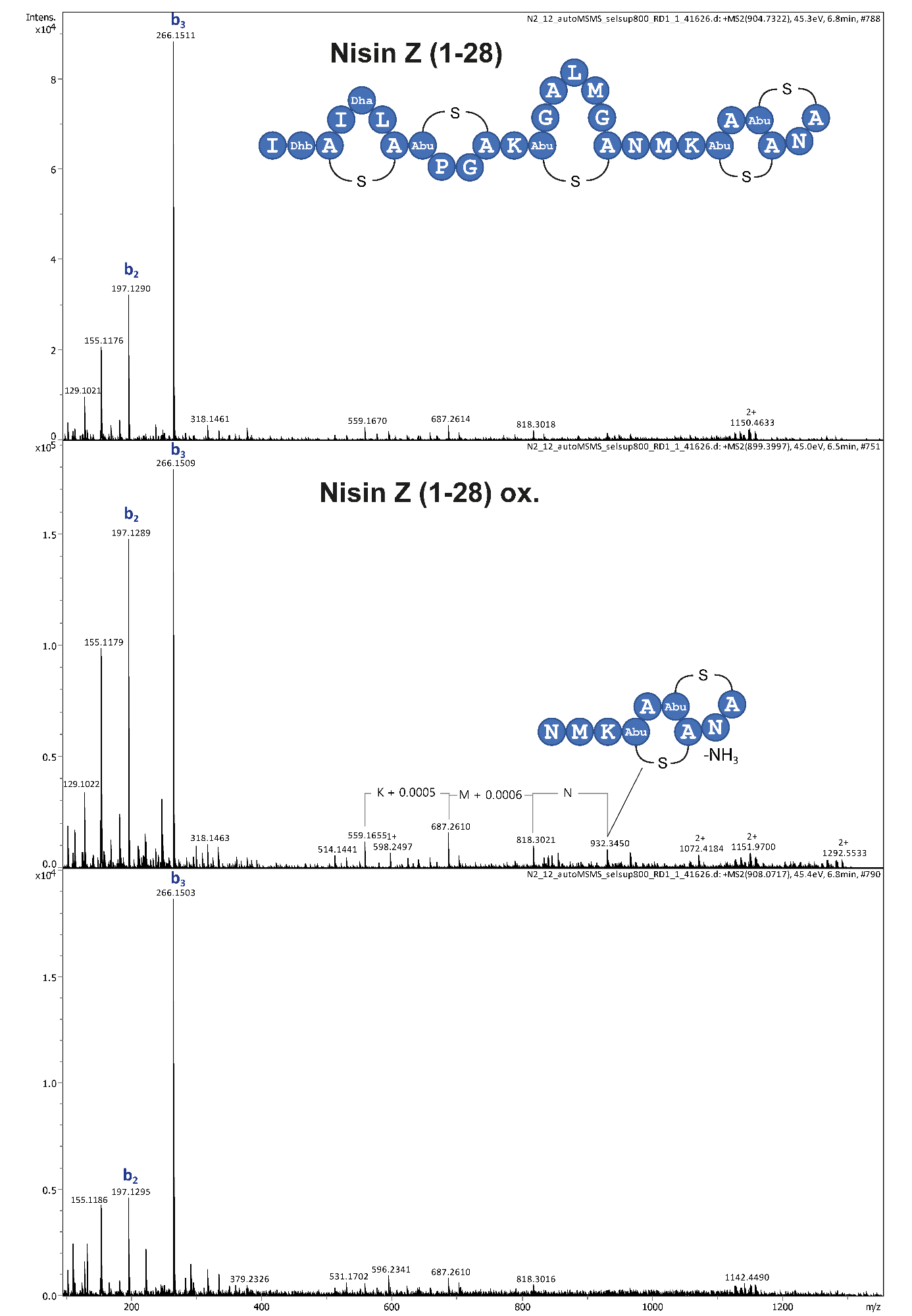 |
| --- | --- |
| B |  |
| C |  |

**Figure S11.** MS/MS spectra of the main degradation products of nisin Z at the [M+3H]^3+^. (A) Nisin Z (1-28) (*m/z* 899.3997); (B) Nisin Z (1-28) ox (*m/z* 904.7320) and (C) Nisin Z (1-28) ox (*m/z* 908.0717).
